# Associations between the multitrajectory neuroplasticity of neuronavigated rTMS‐mediated angular gyrus networks and brain gene expression in AD spectrum patients with sleep disorders

**DOI:** 10.1002/alz.14255

**Published:** 2024-09-26

**Authors:** Weina Yao, Xinle Hou, Huijuan Zhou, Shengqi You, Tingyu Lv, Haifeng Chen, Zhiyuan Yang, Chang Chen, Feng Bai

**Affiliations:** ^1^ Department of Neurology Zhongnan Hospital of Wuhan University Wuhan China; ^2^ Department of Neurology, Nanjing Drum Tower Hospital, Affiliated Hospital of Medical School Nanjing University Nanjing China; ^3^ Department of Neurology, Nanjing Drum Tower Hospital Clinical College of Traditional Chinese and Western Medicine Nanjing University of Chinese Medicine Nanjing China; ^4^ School of Elderly Care Services and Management Nanjing University of Chinese Medicine Nanjing China; ^5^ Geriatric Medicine Center, Taikang Xianlin Drum Tower Hospital, Affiliated Hospital of Medical School Nanjing University Nanjing China; ^6^ Institute of Geriatric Medicine Medical School of Nanjing University Nanjing China

**Keywords:** Alzheimer's disease, gene expression, neural network, sleep disorder, transcription‐neuroimaging association analysis

## Abstract

**INTRODUCTION:**

The multifactorial influence of repetitive transcranial magnetic stimulation (rTMS) on neuroplasticity in neural networks is associated with improvements in cognitive dysfunction and sleep disorders. The mechanisms of rTMS and the transcriptional‐neuronal correlation in Alzheimer's disease (AD) patients with sleep disorders have not been fully elucidated.

**METHODS:**

Forty‐six elderly participants with cognitive impairment (23 patients with low sleep quality and 23 patients with high sleep quality) underwent 4‐week periods of neuronavigated rTMS of the angular gyrus and neuroimaging tests, and gene expression data for six *post mortem* brains were collected from another database. Transcription‐neuroimaging association analysis was used to evaluate the effects on cognitive dysfunction and the underlying biological mechanisms involved.

**RESULTS:**

Distinct variable neuroplasticity in the anterior and posterior angular gyrus networks was detected in the low sleep quality group. These interactions were associated with multiple gene pathways, and the comprehensive effects were associated with improvements in episodic memory.

**DISCUSSION:**

Multitrajectory neuroplasticity is associated with complex biological mechanisms in AD‐spectrum patients with sleep disorders.

**Highlights:**

- This was the first transcription‐neuroimaging study to demonstrate that multitrajectory neuroplasticity in neural circuits was induced via neuronavigated rTMS, which was associated with complex gene expression in AD‐spectrum patients with sleep disorders.
- The interactions between sleep quality and neuronavigated rTMS were coupled with multiple gene pathways and improvements in episodic memory.
- The present strategy for integrating neuroimaging, rTMS intervention, and genetic data provide a new approach to comprehending the biological mechanisms involved in AD.

## 1 BACKGROUND

Alzheimer's disease (AD) is a complex neurodegenerative disease that is characterized by progressive cognitive impairment with severe loss of episodic memory.^1^, ^2^ The sleep status has been significantly related to cognitive impairment in studies of animal models and humans,^3^, ^4^ and sleep disorders have been postulated to be potential risk factors for AD progression.^5^

AD is characterized by a long preclinical period of changes in brain function before substantial cognitive symptoms appear.^6^ Alterations in the default mode network (DMN) are a classic neuroimaging marker for AD.^7^ The left angular gyrus is a core node of the DMN and a vulnerable region in AD patients and is associated with Aβ burden.^8^, ^9^ The steady state of the angular gyrus network primarily supports the function of more advanced cognitive processes, including episodic memory. A recent study combining tractography and microdissection of the angular gyrus wiring diagram revealed its highly distributed connected structure, including connections with the frontal lobe through dorsal and ventral long‐range association fibers (i.e., the angular gyrus anterior network); with the parietal, occipital, and temporal lobes through an abundance of short‐range (including U‐shaped) association fibers (i.e., the angular gyrus posterior network); and with the thalamus, putamen, midbrain, and pons through projection fibers (i.e., the angular gyrus downward network).^10^

Repetitive transcranial magnetic stimulation (rTMS) interventions in AD patients have attracted widespread attention.^11^, ^12^ The high‐frequency rTMS over the left angular gyrus can improve episodic memory and angular gyrus connectivity in AD‐spectrum patients.^13^, ^14^, ^15^ Several rTMS intervention studies have also shown that sleep disorders may have an intrinsic effect on cognitive improvement.^16^, ^17^ Interestingly, a recent study of patients with obstructive sleep apnea revealed abnormal neural activity in the left angular gyrus and impaired cognitive function, with the former possibly being the underlying neural mechanism of the latter.^18^ One of the hallmarks of AD is disrupted iron homeostasis leading to abnormal iron deposition in brain tissue, and a large sample study of 773 participants with obstructive sleep apnea showed increased iron levels in the left angular gyrus.^19^ Therefore, observation of the functional activities and changes in the angular gyrus subnetworks under the dual influence of sleep and rTMS‐targeted intervention can elucidate the underlying mechanism of action on cognitive function in AD‐spectrum patients.

Neuroimaging genetic studies have demonstrated that genetic variation has a lasting impact on the functioning of the brain and is associated with behavior and AD susceptibility.^20^ Single genetic brain–behavior interactions involving GSK3β polymorphisms, apolipoprotein E (APOE) ε4 allele, COMTVal158Met, and ACE D allele were observed in mild cognitive impairment (MCI) and AD patients.^21^, ^22^, ^23^, ^24^, ^25^, ^26^ Our group investigated that the combined effect of tau protein pathway genes was shown to disrupt the topology of the cortico–cerebellar loop.^27^ However, these single‐gene or single‐pathway level studies may have been less accurate and informative than studies based on a whole‐brain transcriptional level.^27^, ^28^ Therefore, the spatial correlation analyses across samples or regions to investigate the relationship between brain gene expression and neuroimaging features of major psychiatric disorders were with great success.^29^, ^30^ However, no transcriptional–neuroimaging correlation studies have been performed in AD‐spectrum patients with sleep disorders.

rTMS may modulate the mRNA expression levels of target genes in AD through its effects on neural plasticity and sleep.^31^, ^32^ This modulation of mRNA expression can lead to a decrease in the production of toxic proteins and an increase in neuronal survival, which may be one of the mechanisms by which rTMS is effective in treating AD. Therefore, we propose that whole‐brain‐level gene expression is associated with the neuroplasticity of neural networks via left angular gyrus‐navigated rTMS in AD‐spectrum patients with different sleep statuses. Here, gene expression data were derived from six *post mortem* brains provided by the Allen Human Brain Atlas (AHBA).^33^ Then, we identified brain gene expression patterns associated with changes in angular gyrus subnetworks in patients on the AD spectrum. Ultimately, the identified genes were performed to identify possible underlying mechanisms and characterize their functions.

## 2 MATERIALS AND METHODS

### 2.1 Participant characteristics

**FIGURE 1 alz14255-fig-0001:**
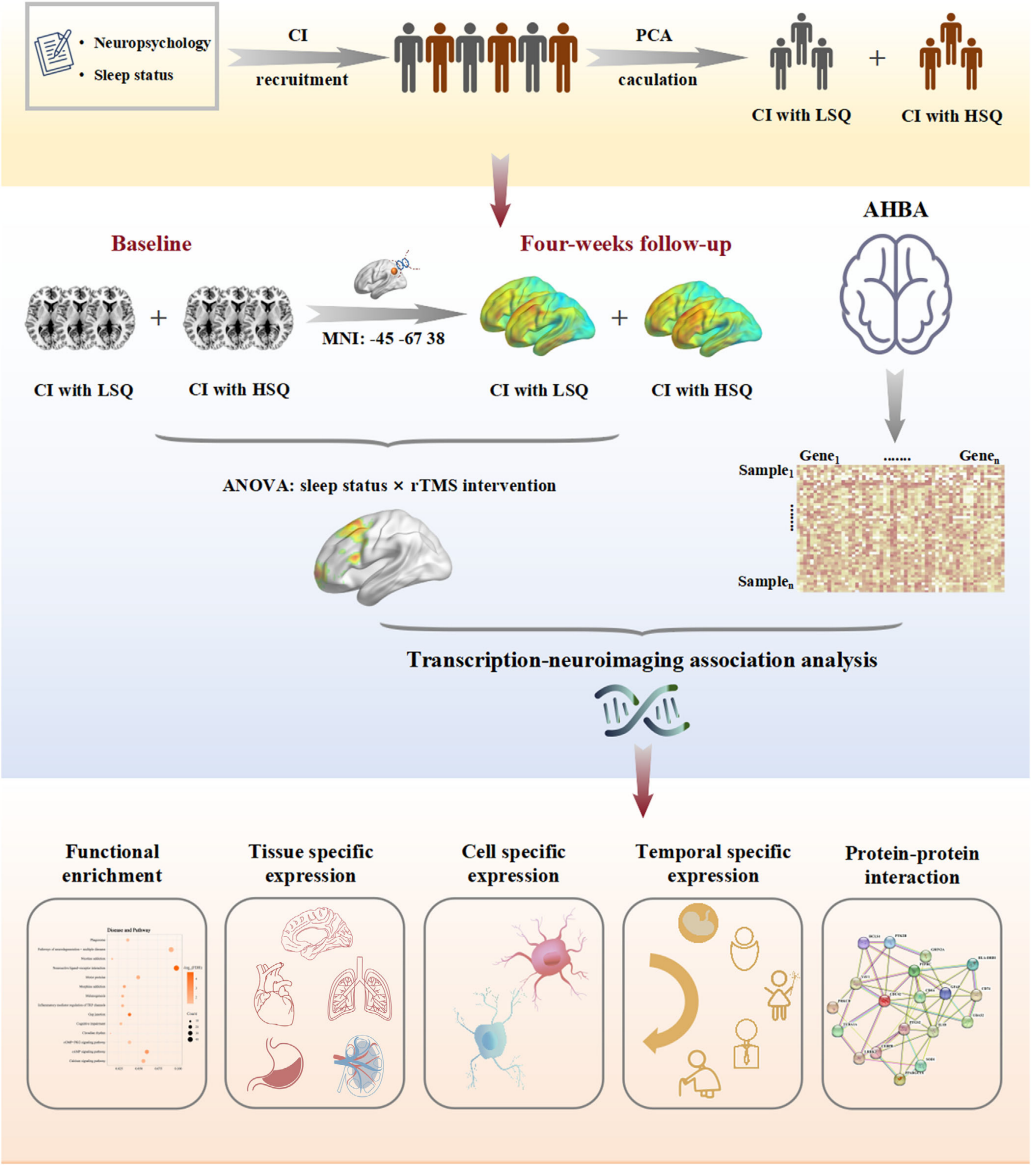
Summary of the study design and the analysis framework. The three main categories included (A) grouping of subjects; (B) a 4‐week period of neuronavigated rTMS treatment and longitudinal fMRI scans; and (C) transcriptional–neuroimaging correlation analysis, which revealed genes associated with functional enrichment, tissue‐specific expression, cell‐specific expression, temporal‐specific expression, and protein‒protein interactions. fMRI, functional magnetic resonance imaging; rTMS, repetitive transcranial magnetic stimulation.

The analysis framework is outlined in Figure 1. A total of 46 elderly participants with cognitive impairment were recruited from the Neurology Department of Drum Tower Hospital of Medical School, Nanjing University. These AD‐spectrum patients included 23 cognitive impairment patients with low sleep quality (CI+LSQ) and 23 cognitive impairment patients with high sleep quality (CI+HSQ) who were matched for sex, age, and education. All participants underwent a complete neurological evaluation, standard laboratory tests, neuroimaging examination, and an extensive battery of neuropsychological assessments. The participants included MCI and AD patients. AD patients were diagnosed based on cerebrospinal fluid pathology or other imaging biomarkers according to the National Institute of Neurological and Communicative Disorders and Stroke and the AD and Related Disorders Association (NINCDS‐ADRDA).^34^ MCI was diagnosed according to the following criteria based on the recommendations of previous studies:^35^, ^36^, ^37^ (i) memory complaint confirmed by the subject and/or an informant; (ii) objective cognitive performance documented by Auditory Verbal Learning Test‐delayed recall scores below or equal to 1.5 standard deviations (SDs) of education and age‐adjusted norms; (iii) Clinical Dementia Rating score = 0.5; (iv) Mini‐Mental State Examination (MMSE) scores ≥24; and (v) symptoms not sufficient for a diagnosis of dementia. The exclusion criteria were as follows (i) other diseases that may cause memory decline, such as cerebrovascular disease, epilepsy, Parkinson's disease, brain tumor, and traumatic brain injury; (ii) major depression (Hamilton Depression Rating Scale [HAMD] ≥ 17) or anxiety (Hamilton Anxiety Rating Scale [HAMA] ≥ 21), schizophrenia, or other mental illness; (iii) severe systemic diseases such as heart failure; (iv) other contraindications for MRI or rTMS; and (v) other primary diseases that cause insomnia, such as apnea syndrome and restless leg syndrome. In addition to these exclusion criteria, none of the patients were taking any cognitive‐improving medications, which would not have been a confounding factor affecting brain function. We simplified the sleep quality scales into a new variable for assessing sleep quality via principal component analysis (PCA), which included the Insomnia Severity Index (ISI), Athens Insomnia Scale (AIS), Pittsburgh Sleep Quality Index (PSQI), Self‐rating Scale of Sleep (SRSS) and Epworth Sleepiness Scale (ESS). For grouping, variables below and above the median were categorized as CI+LSQ and CI+HSQ, respectively. In accordance with differences in sleep quality, 46 patients were categorized into two groups and underwent the entire experimental procedure. The study was approved by the Ethics Committee of Nanjing Drum Tower Hospital, and written informed consent was obtained from all patients before enrolment.

**RESEARCH IN CONTEXT**

1. **Systematic review**: The multifactorial influence of neuroplasticity in neural networks via neuronvigated repetitive transcranial magnetic stimulation (rTMS) is associated with changes in cognitive dysfunction. However, its mechanism of action and transcriptional‐neuronal correlation in Alzheimer's disease (AD) patients with sleep disorders have not been fully elucidated.
2. **Interpretation**: A total of 46 elderly participants with cognitive impairment (23 patients with low sleep quality and 23 patients with high sleep quality) were recruited, and the data included clinical data, 4‐week periods of left angular gyrus‐navigated rTMS treatment, neuroimaging data, and another six *post mortem* brains from neurotypical individuals in the Allen Human Brain Atlas associated with gene expression data. We explored the spatial information available about the interactions between sleep status and rTMS intervention and its effect on cognitive dysfunction and the possible biological mechanisms (i.e., functional enrichment, specific expression, behavioral correlation, and protein‒protein interaction analyses). Neuroimaging genetic evidence has indicated that the multitrajectory neuroplasticity of neural circuits is provoked by neuronucleated rTMS, which may also be associated with the complex biological mechanisms involved in AD‐spectrum patients with sleep disorders.
3. **Future directions**: The transcription‐neuroimaging study revealed that cognitive impairment and sleep disorders may occur through complex multigene and multipathway mechanisms and may serve as candidate genes for exploring the molecular mechanisms of neurodegeneration. These findings provide a new approach to comprehending the biological mechanisms of AD.

### 2.2 Neuronavigated rTMS and neuropsychological assessment

Neuronavigated rTMS was performed using a commercially available magnetic stimulator (CCY‐IV model; Yirid, Inc., Wuhan, China) with a 70 mm figure‐8 coil. Because rTMS cannot directly intervene in the hippocampus, the therapeutic target of the study was calculated in another AD spectrum cohort from our center via seed‐based functional connectivity analysis using the left hippocampus as a seed.^14^ Finally, the target region was localized in the left angular gyrus (Montreal Neurological Institute: ‐45, ‐67, 38) due to the significant differences in its functional connectivity among the healthy control, MCI, and AD participants. The stimulation target was defined as a sphere with a radius of 6 mm centered on the coordinates, which was then individually transformed into T1 space for each participant using the inverse matrix generated during the T1 segmentation process in the Statistical Parametric Mapping analysis package (https://www.fil.ion.ucl.ac.uk/spm/software/spm12/, SPM12) and TMStarget software. Stimulation targets for each individual were entered into the navigation system (Visor 2.0, Advanced Neuro Technologies, Enschede, The Netherlands). The stimulation parameters used were as follows: 20 Hz stimulation frequency, 2 s duration, 1600 stimulations per group (including 40 columns per group and 40 stimulations per column), and 28 s intervals without stimulation. The stimulus intensity was set to 100% of each patient's resting motor threshold, which was the minimum stimulus intensity required to elicit a contraction of the right short adductor muscle (at least five consecutive pulses with a contraction threshold of 50 µV). rTMS treatment consisted of a total of 20 stimulation sessions lasting 20 min over 4 weeks (Monday through Friday, weekends off). Dynamic real‐time monitoring was performed during the entire treatment process for precise control of the position. Posttreatment fMRI and neuropsychological assessments were collected at the end of the 4‐week treatment period.[Fig alz14255-fig-0001]

Before and after rTMS treatment, all subjects underwent a battery of neuropsychological tests, including cognitive domains and sleep tests assessing cognitive status and sleep, respectively. The neuropsychological tests were used to assess general and specific cognitive domains. General cognitive function was assessed using the Chinese version of the MMSE and Montreal Cognitive Assessment (MoCA). The specific cognitive domains included episodic memory (Auditory Verbal Learning Test‐immediate recall (AVLT‐IR), AVLT‐short recall [AVLT‐SR], AVLT‐long recall [AVLT‐LR], Wechsler Memory Scale Visual Reproduction delayed recall [VR‐DR]), processing speed, executive function, and language function.

In addition, all participants completed a series of self‐administered questionnaires to evaluate sleep status. Specifically, the ISI measures the severity of insomnia symptoms. The AIS is a tool utilized to assess insomnia incidence. The PSQI is a retrospective self‐report questionnaire that assesses subjective sleep quality over the previous month. The SRSS is a questionnaire used to evaluate sleep quality over the past month. Sleepiness during the day was assessed using the ESS.^38^

### 2.3 Functional MRI acquisition and preprocessing

All participants underwent functional MRI (fMRI) scans at baseline and during the 4‐week follow‐up period. fMRI data were acquired using a Philips 3.0T scanner (Philips Medical Systems). After treatment, resting‐state fMRI data were acquired using gradient echo‐planar imaging with the following parameters: repetition time (TR)  =  2000 ms; echo time (TE)  =  30 ms; thickness  =  4.0 mm; flip angle (FA)  =  90°; acquisition matrix  =  64 × 64; slice number  =  35; and spatial resolution  =  3 × 3 × 3 mm^3^. All subjects had their eyes closed during scanning. The fMRI data were preprocessed in the MATLAB R2013b environment within the Data Processing Assistant for Resting‐state fMRI v2.2 (DPARSF). To mitigate the effects of the instrument on participants during the initial scan, the first 10 points of the time series were removed. Preprocessing included slice‐timing, realigning, normalization, spatial smoothing, detrending, nuisance regression and filtering. Participants with excessive head movements and excessive rotations were excluded from the study.

### 2.4 Sleep status × rTMS intervention interaction analysis and behavioral significance

The rTMS stimulation target of the left angular gyrus (Montreal Neurological Institute [MNI]: ‐45, ‐67, 38) was used as the seed, and a sphere with a radius of 6 mm centered on the MNI coordinates was selected; this sphere was used to calculate the average signal change in the region of interest for all patients. Pearson correlation analysis was subsequently performed between the average signal change in the left angular gyrus and the whole‐brain voxel time series. Next, a Z‐matrix was generated by converting the Pearson correlation coefficient into a z value close to the normal distribution using Fisher's z‐transformation (*z* = 0.5 × ln(1+r)/(1‐r)). These procedures were implemented in the REST Toolkit (https://restfmri.net). To remove the possible effects of head motion and white matter and cerebrospinal fluid signals on the results, six head motion parameters and the mean time series of white matter and cerebrospinal fluid signals were introduced as covariates. A mixed analysis of variance (ANOVA) was performed, with a within‐subject repeated factor (rTMS intervention time points: baseline and follow‐up) and an across‐subject factor (groups: CI+LSQ and CI+HSQ) with the subject as a nested random variable within the group. This analysis was performed on the four groups using the SPM12 toolkit (http://www.fil.ion.ucl.ac.uk/spm). Interaction changes were explored using post hoc tests (Monte Carlo simulation, corrected *p* < 0.05, and a cluster size of 6165 mm^3^). The average functional connectivity values within the sleep status × rTMS intervention interaction mask were extracted from each subject's images, subsequently, further direct comparisons of the change estimates between the four groups were conducted.

To investigate the behavioral significance of sleep status × rTMS intervention on angular gyrus networks, Pearson correlation analyses were utilized to explore whether voxelwise interactions were correlated with episodic memory performance in CI+LSQ and CI+HSQ subjects (*p* < 0.05 was considered significant). To consider the comprehensive efficacy of multiple episodic memory scales, including the AVLT‐IR, AVLT‐SR, AVLT‐LR, and VR‐DR, the raw scores for each test for each subject were converted into *z*‐scores with reference to the means and standard deviations of all the subjects; we then calculated the weighted scores by summing the *z*‐scores of the four individual tests. In addition, to avoid confounding factors that may have affected the results, the effects of age, education, sex, and sleep status were included as covariates of interest.

### 2.5 Brain gene expression data processing

Brain gene expression data were obtained from the downloadable AHBA dataset (http://www.brain‐map.org).^33^, ^39^ The dataset was derived from six human *post mortem* donors. The original expression data of more than 20,000 genes in 3702 spatially distinct brain tissue samples were processed using a newly proposed pipeline.^40^

### 2.6 Identification of genes associated with sleep status × rTMS intervention at the whole‐brain voxel level

After these processing procedures, we obtained normalized expression data for 15,633 genes from 3121 tissue samples. Since our neuroimaging analyses were performed within the sleep status × rTMS intervention‐interacting brain regions, we further restricted our analyses to samples from that mask, with a final sample gene matrix of 3121 × 1119. The number of remaining genes in the processing step is shown in the Supporting Information.

### 2.7 Transcription‐neuroimaging association analysis

The following spatial correlation analyses were used to identify genes associated with functional changes in the brain regions affected by the sleep status × rTMS intervention interaction. To determine the functional changes associated with the interacting brain region, we defined the brain region and extracted the average z values of the spherical endosomal voxels from the Z‐matrix. Cross‐sample Pearson correlations between gene expression and z values were then performed genetically to identify genes with expression levels associated with changes in participants' brain function. Multiple comparisons were corrected using the Benjamini–Hochberg false discovery rate (FDR‐BH) method (*p* < 0.001). To further test whether the number of identified genes was significantly greater than the number of randomized genes, we performed a spatially constrained permutation test to determine the significance of our results.

### 2.8 Gene enrichment analysis

The genes associated with the sleep status × rTMS intervention interaction were analyzed via a set of enrichments. Functional annotation was performed by Gene Ontology (GO) biological process and Kyoto Encyclopedia of Genes and Genomes (KEGG) (identifying genes associated with specific biological pathways) database enrichment analysis of the genes implemented in the clusterProfiler R package. GO was utilized to assess the biological functions of the genes, including molecular functions, biological processes, and cellular components. Pathways and disease databases were used to assess biological pathways and associated diseases. The details can be found in the Supporting Information. Subsequently, we used the online Tissue‐Specific Expression Analysis (TSEA) tool (http://genetics.wustl.edu/jdlab/tsea/) and the Cell Type‐Specific Expression Analysis Tool (http://genetics.wustl.edu/jdlab/csea‐tool‐2/)^41^ for tissue‐, cell type‐ and time‐specific expression analyses. These expression analyses assisted in identifying specific tissues, cortical cell types, and developmental stages in which genes associated with interaction regions were overexpressed. Specificity index probabilities (*p*SIs = 0.05 and 0.01) were used to determine the likelihood of gene‐specific expression. A corrected *p*‐value of 0.05 was used to correct for multiple testing using FDR‐BH.

### 2.9 Protein‒protein interaction analysis

Protein‒protein interaction (PPI) data were obtained using the STRING database v11.0 (http://string‐db.org), for which the minimum required interaction score was 0.7. A PPI network was constructed, and hub genes related to sleep status × rTMS intervention were identified. The PPI confidence interaction score was set at ≥ 0.9, where the top 10% of nodes in the PPI network were defined as hub genes; a list of the hub genes can be found in the Supporting Information. Furthermore, the spatial–temporal expression trajectories of representative hub genes were characterized utilizing the Human Brain Transcriptome Database (http://hbatlas.org/).

### 2.10 Statistical analysis

All the statistical analyses were performed using SPSS 22.0 software (SPSS, Inc., Chicago, IL, USA) with the threshold set at *p* < 0.05. Differences in demographic characteristics, neuropsychological test scores, and sleep status between the CI+LSQ group and the CI+HSQ group were assessed by the paired samples Wilcoxon signed‐rank test followed by multiple comparisons using the Sidak correction. The nonparametric Mann‒Whitney *U*‐test was used for nonnormally distributed data to compare the differences between the two groups before and after treatment and was then corrected for multiple comparisons using the Sidak correction.

## 3 RESULTS

### 3.1 Demographic and clinical characteristics

**FIGURE 2:**
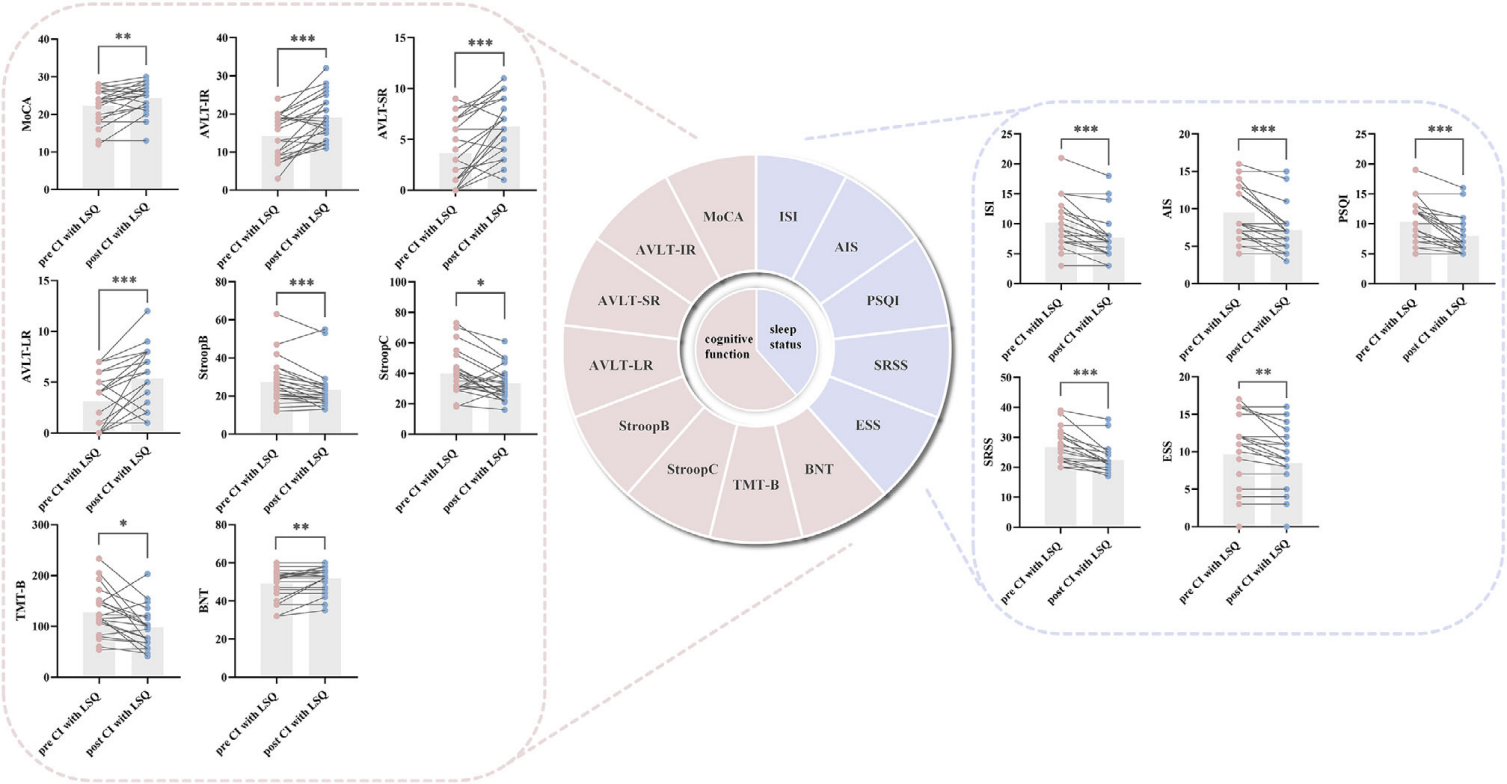
After 4 weeks of neuronavigated rTMS treatment, the CI+LSQ group exhibited significant improvements in performance on the neuropsychological (left side) and sleep tests (right side) compared to before treatment. Thresholds were set at a corrected *p* < 0.05. CI+LSQ, cognitive impairment patients with low sleep quality; rTMS, repetitive transcranial magnetic stimulation.

The demographic characteristics and neuropsychological test results of each group are listed in Table 1. There were no significant differences in terms of age, education level, or sex ratio between the CI+LSQ and CI+HSQ groups (*p* > 0.05). (i) Within group: after 4 weeks of neuronavigated rTMS treatment, the CI+LSQ group achieved more significant improvements in performance on the neuropsychological and sleep tests. Specifically, compared with those in the baseline period, the CI+LSQ group demonstrated significant improvements in general cognition (i.e., MoCA score), episodic memory (i.e., AVLT‐IR, AVLT‐SR, and AVLT‐LR), processing speed (i.e., Stroop B), executive function (i.e., Stroop C and Trail Making Test [TMT]‐B), language function (i.e., Boston Naming Test [BNT]) and sleep status (i.e., ISI, AIS, PSQI, SRSS, and ESS) (Figure 2). However, the CI+HSQ group exhibited significant differences only in episodic memory (i.e., AVLT‐IR, AVLT‐SR, AVLT‐LR, and VR‐DR) and language function (i.e., BNT) (Figure SI 1). (ii) Between groups: we further compared the differences in values before and after treatment among the different groups. We found that more significant longitudinal improvements in sleep quality and executive function were observed in the CI+LSQ group than in the CI+HSQ group. The details of the correlations between the improvement in sleep quality and the improvement in cognitive dysfunction after rTMS treatment are provided in the Supporting Information (Table SI 1).

**TABLE 1:** Demographic and neuropsychological data between and within groups.

	Pre		Post		*p*‐value (pre vs. post)		*p*‐value (Δ)
Items	CI+LSQ (*n* = 23)	CI+HSQ (*n* = 23)	CI+LSQ (*n* = 23)	CI +HSQ (*n* = 23)	CI +LSQ (*n* = 23)	CI+HSQ (*n* = 23)	(ΔCI+LSQ vs. ΔCI +HSQ)
**Demographics**							
Age (years)	68.20 ± 8.07	68.60 ± 6.14	–	–	–	–	–
Education (years)	11.96 ± 3.13	11.39 ± 2.64	–	–	–	–	–
Sex (male/female)	(7/16)	(11/12)	–	–	–	–	–
**General cognition**							
MMSE	26.96 ± 3.31	26.04 ± 3.05	27.74 ± 2.51	27.09 ± 2.81	0.06164	0.10350	0.75754
MoCA	22.22 ± 4.54	22.30 ± 3.71	24.35 ± 3.96	23.22 ± 3.94	0.00402^**^	0.17218	0.18152
**Episodic memory**							
AVLT‐IR	14.17 ± 5.75	13.04 ± 4.76	19.00 ± 5.89	15.57 ± 4.50	0.0001^***^	0.01205^*^	0.12205
AVLT‐SR	3.65 ± 3.02	3.39 ± 2.74	6.26 ± 2.82	4.83 ± 3.14	0.00004^***^	0.00063^***^	0.06532
AVLT‐LR	3.13 ± 2.58	3.13 ± 2.94	5.35 ± 2.96	4.52 ± 3.53	0.00026^***^	0.00591^**^	0.25548
VR‐DR	6.48 ± 4.09	4.26 ± 3.70	8.39 ± 4.14	6.13 ± 4.41	0.22161	0.00468^**^	0.96232
**Processing speed**							
TMT‐A (raw score)	76.26 ± 56.26	88.43 ± 39.06	64.26 ± 41.80	78.35 ± 34.57	0.38843	0.21059	0.90961
Stroop A (raw score)	27.65 ± 22.17	24.74 ± 6.68	26.13 ± 28.12	24.96 ± 7.41	0.50490	0.88714	0.46678
Stroop B (raw score)	27.35 ± 11.51	28.65 ± 10.03	23.17 ± 10.60	27.96 ± 8.69	0.00096^***^	0.72182	0.08360
**Executive function**							
Stroop C (raw score)	39.83 ± 14.54	37.57 ± 11.39	33.43 ± 10.75	43.96 ± 17.09	0.01073^*^	0.10679	0.00972^**^
TMT‐B (raw score)	144.52 ± 92.05	171.26 ± 96.05	101.74 ± 42.37	200.22 ± 149.82	0.01237^*^	0.16078	0.00678^**^
**Language function**							
BNT	49.22 ± 8.13	50.13 ± 6.90	51.83 ± 6.83	51.91 ± 4.50	0.00804^**^	0.00576^**^	0.48144
**Sleep status**							
ISI	10.22 ± 3.99	2.57 ± 1.62	7.70 ± 3.30	2.39 ± 1.44	0.000004^***^	0.16186	0.00002^***^
AIS	9.52 ± 3.57	2.83 ± 1.59	7.17 ± 3.05	2.65 ± 1.50	0.00002^***^	0.16186	0.00007^***^
PSQI	10.35 ± 3.52	4.09 ± 1.93	8.00 ± 3.06	3.78 ± 1.38	0.00009^***^	0.20017	0.00077^***^
SRSS	26.78 ± 5.73	16.43 ± 1.95	22.39 ± 4.62	15.87 ± 2.05	0.000009^***^	0.08489	0.00010^***^
ESS	9.65 ± 4.97	5.74 ± 3.73	8.48 ± 4.22	5.61 ± 3.63	0.00179^**^	0.32818	0.01076^*^

*Note*: Values are presented as the average ± standard deviation (SD). *p* (pre vs. post) indicates the comparison between pre‐treatment and post‐treatment, which was obtained by the paired samples Wilcoxon signed‐rank test followed by multiple comparisons using Sidak correction. *p* (Δ) values were obtained by the Mann‒Whitney *U*‐test followed by multiple comparisons using Sidak correction, which was used here to compare the differences between the two groups before and after treatment.

Abbreviations: AIS, Athens Insomnia Scale; AVLT, Auditory Verbal Learning test; AVLTH‐IR, AVLTH‐immediate recall; AVLTH‐LR, AVLTH‐long recall (3−5 min vs. 20 min delay); AVLTH‐SR, AVLTH‐short recall; BNT, Boston Naming Test; CI+HSQ, cognitive impairment patients with high sleep quality; CI+LSQ, cognitive impairment patients with low sleep quality; ESS, Epworth Sleepiness Scale.; ISI, Insomnia Severity Index; MMSE, Mini‐Mental State Examination; MoCA, Montreal Cognitive Assessment; PSQI, Pittsburgh Sleep Quality Index; SRSS, Self‐Rating Scale of Sleep; StroopA, B and C, Stroop Color and Word Tests A, B and C; TMT‐A and ‐B, Trail Making Tests‐A and ‐B; VR‐DR, Wechsler Memory Scale, visual reproduction delayed recall.

* Indicates a significant difference between groups: ^*^ *p* < 0.05; ^**^ *p* < 0.01; ^***^ *p* < 0.001.

### 3.2 Sleep status × rTMS intervention ANOVA of left angular gyrus network functional connectivity

**FIGURE 3:**
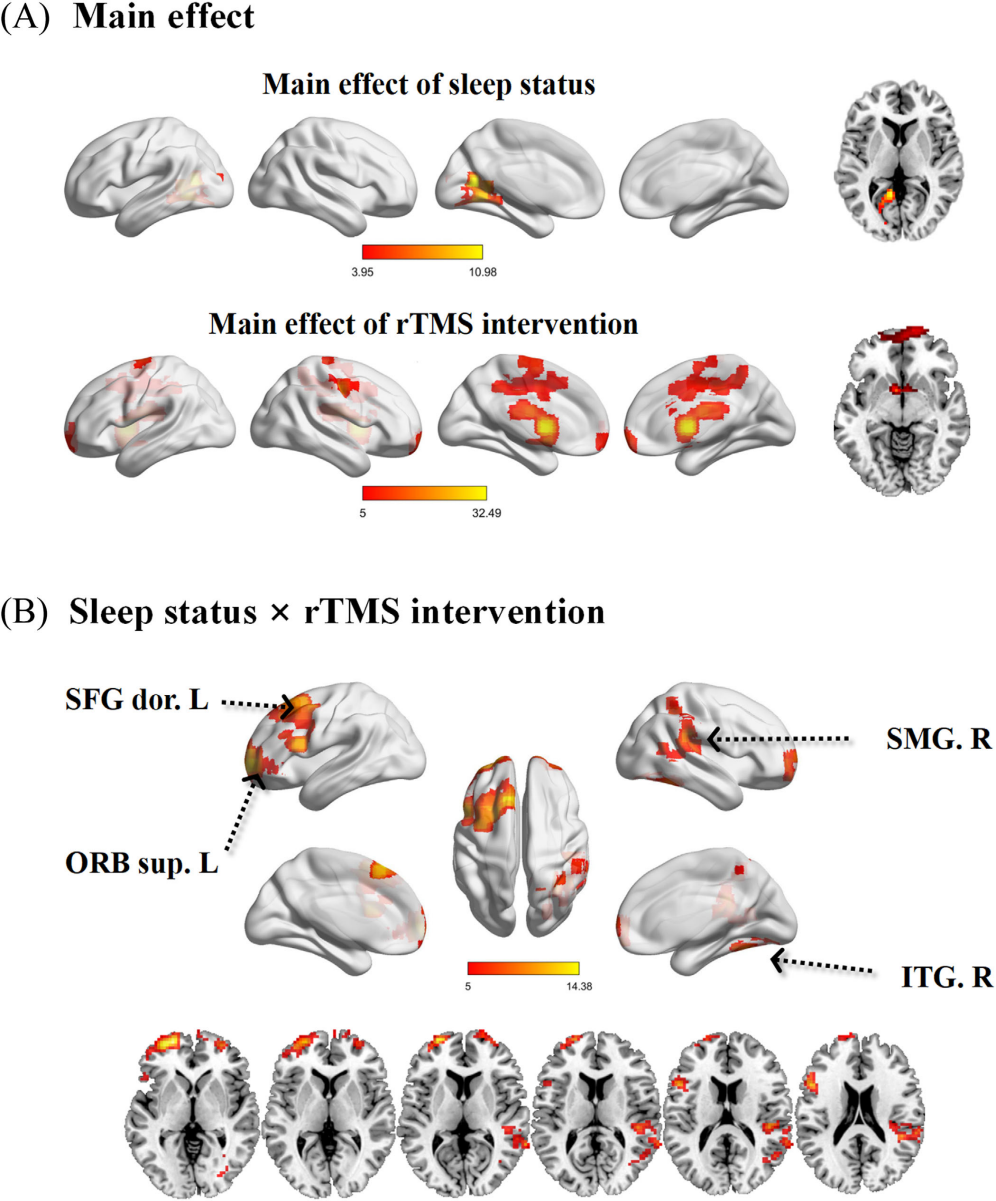
Sleep status × rTMS intervention ANOVA of angular network functional connectivity. (A) Spatial distributions of the main effects of sleep status and rTMS intervention. (B) ANOVA was performed for the angular gyrus‐anterior network (i.e., left superior dorsal/orbital frontal gyrus [SFG dor.L/ORB sup.L]) and the angular gyrus‐posterior network (i.e., right inferior temporal gyrus/right supramarginal gyrus, [ITG.R/SMG.R]). Thresholds were set at a corrected *p* < 0.05, determined by Monte Carlo simulation. ANOVA, mixed analysis of variance; rTMS, repetitive transcranial magnetic stimulation.

The brain function network was constructed via a seed‐based approach for each subject; next, a mixed ANOVA was performed between groups (Figure 3). The results of the ANOVA with sleep status and rTMS intervention as the main effects are shown in Figure 3A. A qualitative visual inspection of angular gyrus‐temporal connectivity revealed that sleep status was associated with the main effect, while the regions along the pathway from the angular gyrus to the hippocampus were related to the main effect of rTMS intervention (i.e., the patterns confirmed that the therapeutic target of the angular gyrus was selected by hippocampal seed‐based functional connectivity analysis). In particular, the patterns of sleep status × rTMS intervention ANOVA interactions and the distribution of brain networks were confirmed in most clusters, including the angular gyrus anterior network (i.e., left superior dorsal/orbital frontal gyrus) and the angular gyrus posterior network (i.e., right inferior temporal gyrus/right supramarginal gyrus) (Figure 3B and Table SI 2). Monte Carlo simulations were utilized to correct for the threshold of *p* < 0.05.

### 3.3 Longitudinal changes in brain network differences before and after rTMS treatment and their behavioral significance

**FIGURE 4:**
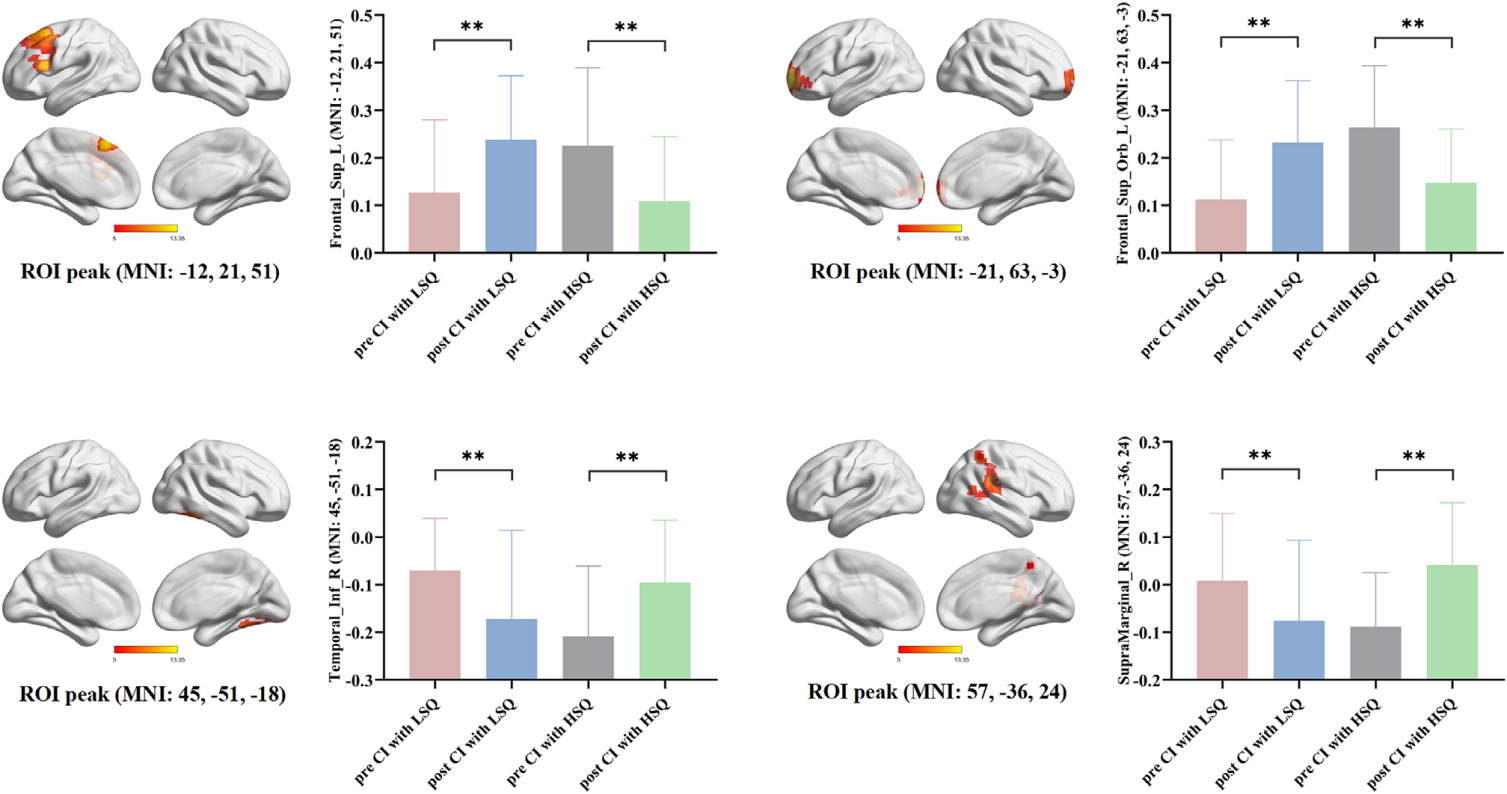
Post hoc analysis of ANOVA interactions. (A) Between groups: completely opposite network action patterns were observed in the two sleep quality groups within the same subnetwork. Namely, compared with those in the baseline period, the CI+LSQ group exhibited linear upward angular gyrus‐anterior network connections and linear downward angular gyrus‐posterior network connections at follow‐up. The CI+HSQ group exhibited the opposite pattern. (B) Between subnetworks: the angular gyrus anterior network (i.e., left superior dorsal/orbital frontal gyrus) and posterior network (i.e., right inferior temporal gyrus/right supramarginal gyrus) may have antagonistic effects. Thresholds were set at a corrected *p* < 0.05, determined by Monte Carlo simulation. ANOVA, a mixed analysis of variance; CI+HSQ, cognitive impairment patients with high sleep quality; CI+LSQ, cognitive impairment patients with low sleep quality; MNI, Montreal Neurological Institute; ROI, region of interest.

Significant differences in brain function changes between the CI+LSQ group and CI+HSQ group before and after rTMS treatment indicated that the angular gyrus networks exhibited distinct variability in longitudinal characteristics within groups (determined by Monte Carlo simulation, *p* < 0.05; Figure 4, and Table SI 3). Overall, the angular gyrus anterior network and posterior network may have mutually antagonistic effects. Compared with those in the baseline period, the CI+LSQ group exhibited linear upward angular gyrus anterior network connections (i.e., increased connectivity in the left superior dorsal/orbital frontal gyrus) and linear downward angular gyrus posterior network connections (i.e., decreased connectivity in the right inferior temporal gyrus/right supramarginal gyrus) at follow‐up (determined by Monte Carlo simulation, *p* < 0.05). Interestingly, these patterns in functional connectivity were completely reversed in the CI+HSQ group.

**FIGURE 5:**
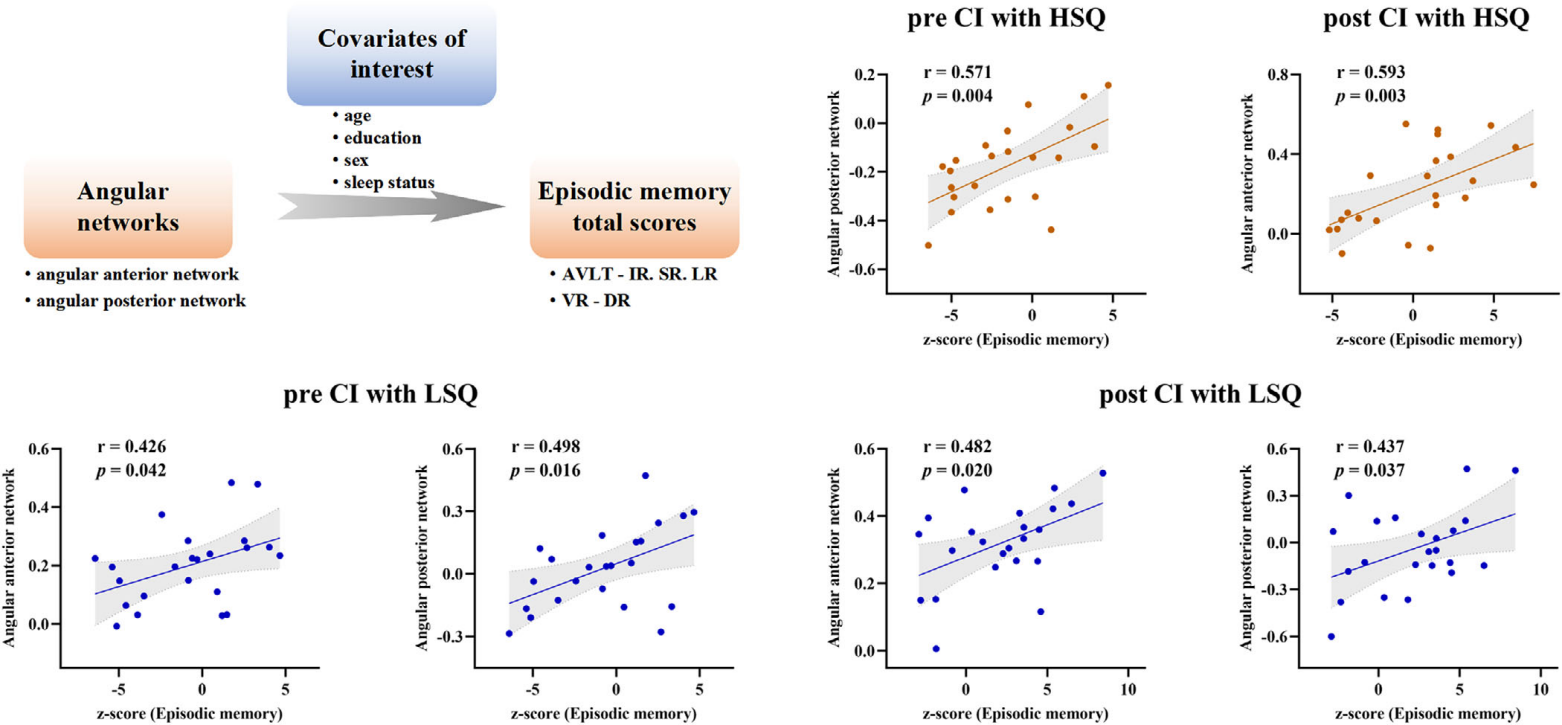
Behavioral significance of ANOVA interactions and neuropsychology and sleep. In the CI+LSQ group, there was a positive correlation between the connectivity of the angular gyrus‐anterior network and episodic memory both at baseline (*r* = 0.426, *p* = 0.042) and after 4 weeks of rTMS treatment (*r* = 0.482, *p* = 0.020); similar associations were detected in the angular gyrus‐posterior network both at baseline (*r* = 0.498, *p* = 0.016) and after 4 weeks of rTMS treatment (*r* = 0.437, *p* = 0.037). In addition, the CI+HSQ group exhibited positive correlations only in the angular gyrus‐posterior network at baseline (*r* = 0.571, *p* = 0.004) and in the angular gyrus‐anterior network at follow‐up (*r* = 0.593, *p* = 0.003). ANOVA, a mixed analysis of variance; CI+HSQ, cognitive impairment patients with high sleep quality; CI+LSQ, cognitive impairment patients with low sleep quality; rTMS, repetitive transcranial magnetic stimulation.

The following process was used to explore the behavioral significance of the ANOVA interactions. As shown in Figure 5, partial correlations were performed to demonstrate that the intrinsic connectivity strength according to the ANOVA interactions was significantly correlated with episodic memory in these patients. Specifically, CI+LSQ was positively correlated with the connectivity of the angular gyrus anterior network and episodic memory both at baseline (*r* = 0.426, *p* = 0.042) and after 4 weeks of rTMS treatment (*r* = 0.482, *p* = 0.020), and a similar association was detected in the angular gyrus posterior network both at baseline (*r* = 0.498, *p* = 0.016) and after 4 weeks of rTMS treatment (*r* = 0.437, *p* = 0.037). However, CI+HSQ scores were positively correlated with only the angular gyrus posterior network at baseline (*r* = 0.571, *p* = 0.004) and the angular gyrus anterior network at follow‐up (*r* = 0.593, *p* = 0.003).

### 3.4 Interaction effect of sleep status and rTMS intervention on gene expression

After relabeling and probe selection, we ultimately obtained normalized expression data for 15,633 genes from 3121 samples from the AHBA database. By utilizing transcriptome‐neuroimaging spatial correlation analysis, we identified 1119 genes (Table SI 4) with expression levels significantly correlated with sleep status × rTMS intervention interactions and associated with whole‐brain voxels in all cognitively impaired patients (FDR‐BH corrected, *p* < 0.001). Incidentally, the tests for spatially constrained alignments demonstrated that our results diverged from random results; specifically, none of the 5000 alignments identified more genes than those identified using real data (*p*_perm_ < 0.001).

### 3.5 Gene functional enrichment

**FIGURE 6:**
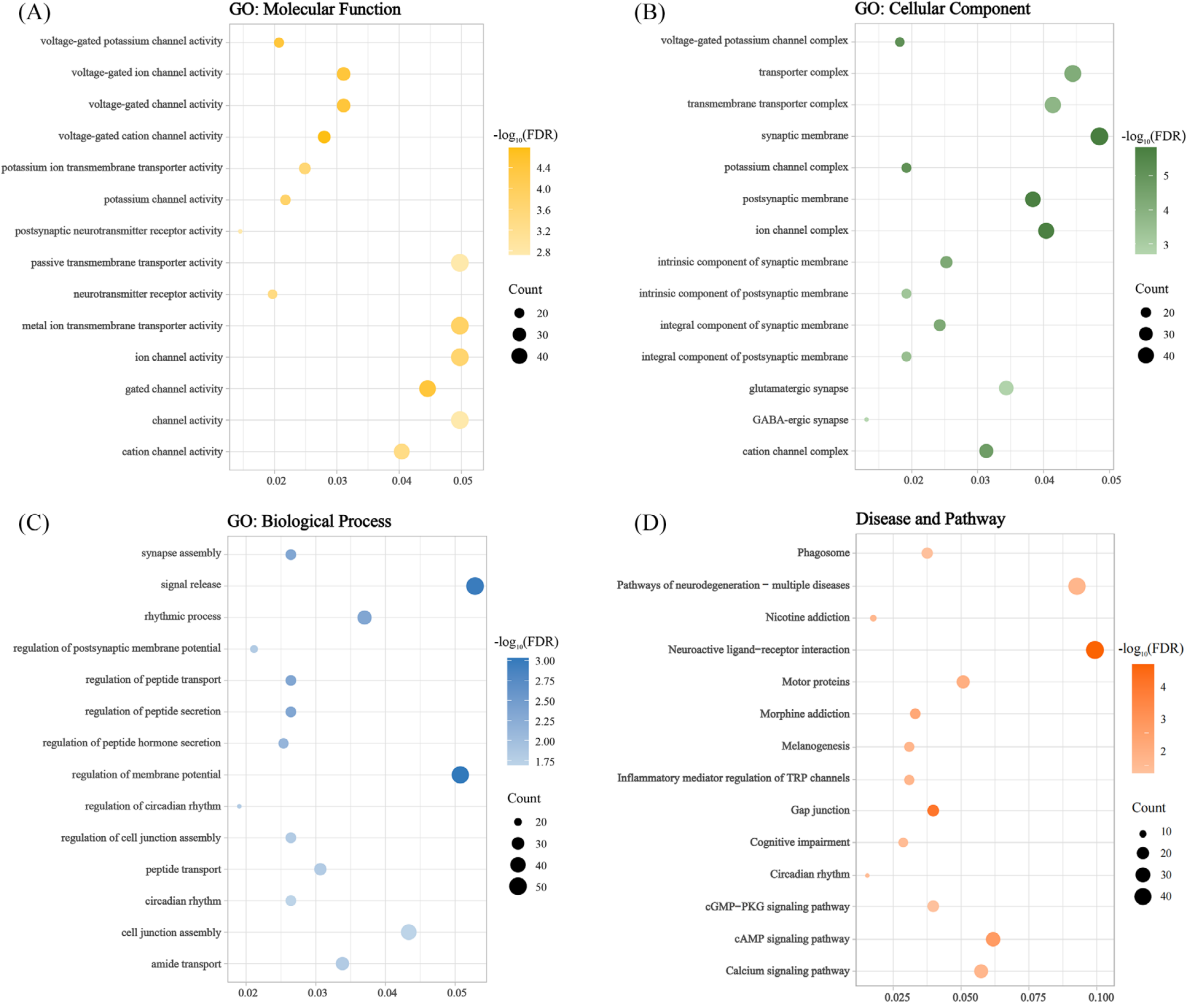
Functional enrichment of sleep status × rTMS intervention interaction‐related genes. GO and KEGG enrichment analysis: (A) GO: molecular function. (B) GO: cellular component. (C) GO: biological process. (D) Disease and pathway (KEGG terms). The *x*‐axis represents the rich factor, and the *y*‐axis represents the item. The rich factor is the ratio of the number of genes annotated to the item to the number of all genes annotated to the item. The bubble size indicates the number of genes overlapping each item, the bubble color indicates the ‐log_10_ (FDR) value, and the *p*‐value was corrected by the FDR‐BH method. FDR, false discovery rate; FDR‐BH, Benjamini–Hochberg false discovery rate; GO, Gene Ontology; KEGG, Kyoto Encyclopedia of Genes and Genomes. GO, Gene Ontology; KEGG, Kyoto Encyclopedia of Genes and Genomes; rTMS, repetitive transcranial magnetic stimulation.

To characterize the biological functions, diseases and pathways associated with sleep status × rTMS intervention interactions in cognitively impaired patients, we performed a functional enrichment analysis using the clusterProfiler software package in R. The results of the gene function enrichment analyses are listed in the Supporting Information: Table SI 5 and shown in Figure 6. In the GO analysis, 1119 genes were found to be enriched in molecular functions (i.e., voltage‐gated ion channel activity, neurotransmitter receptor activity, and metal ion transmembrane transporter activity) (Figure 6A), cellular components (i.e., synaptic membrane, GABAergic synapse, and ion channel complex) (Figure 6B), and biological processes (i.e., synapse assembly, signal release, and peptide transport) (Figure 6C). Regarding diseases, the identified genes were enriched in several psychiatric disorders, including multiple neurodegenerative diseases and cognitive disorders (Figure 6D). As shown by the KEGG pathway analysis, the differentially expressed genes were associated with the cGMP‐PKG signaling pathway, cAMP signaling pathway, and calcium signaling pathway (Figure 6D).

### 3.6 Tissue‐, cell type‐, and temporal‐specific expression

Tissue‐specific expression analysis revealed that some genes were expressed specifically in brain tissue (Figure SI 2a). For the set of genes specifically expressed in brain tissue, we further investigated their specific expression in terms of cortical cell types and developmental stages. Specifically, cortical cell types in multineurons and nerve cells (excitatory neurons, postexcitatory neurons, astrocytes, neural progenitor cells, and oligodendrocyte progenitor cells) were found to express these genes (Figure SI 2b). Time‐specific expression analyses revealed that these genes were expressed during mid‐to‐late development in the hippocampus, amygdala, thalamus, striatum, and cerebellum, whereas they were mainly expressed during pre‐ and mid‐development in the cortex (Figure SI 2C–H).

### 3.7 PPI networks and hub genes

**FIGURE 7:**
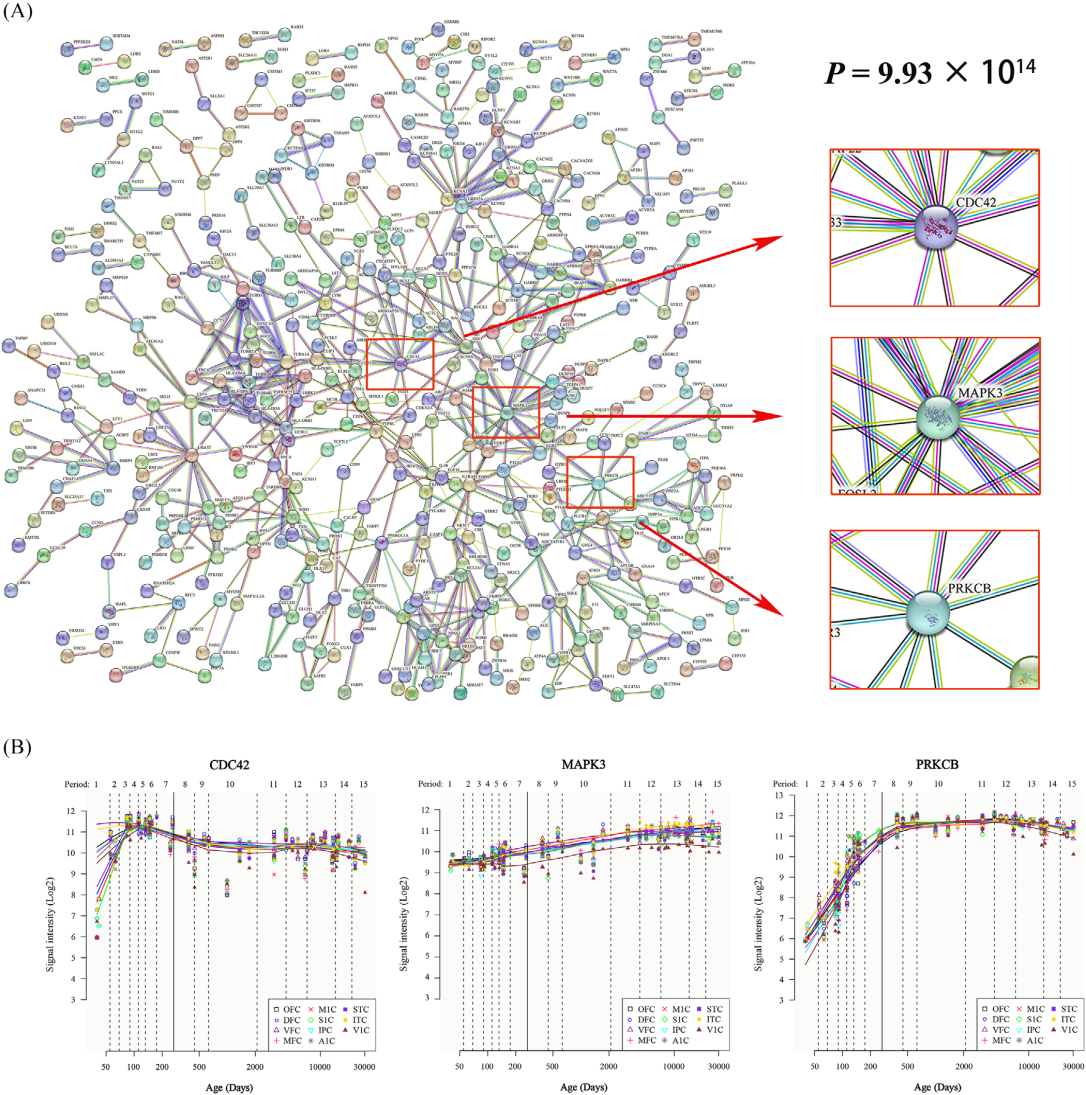
PPI networks were constructed from AD‐spectrum patient sleep status × rTMS intervention interaction‐related genes. (A) PPI network containing 376 genes. Among these genes, CDC42, MAPK3, and PRKCB were the three central genes with the highest degree values. The *p*‐value denotes the statistical significance of the input genes that encode proteins connecting the constructed network. (B) Spatial and temporal expression profiles of CDC42, MAPK3, and PRKCB. The *x*‐axis represents the expression of a specific gene strictly in a specific chronological order to meet the needs of specific differentiation and development stages of cells or individuals. The *y*‐axis represents the signal intensity of genes. The bottom box displays the expression of this gene in neocortical areas at a certain time point. AD, Alzheimer's disease; PPI, protein‒protein interaction; rTMS, repetitive transcranial magnetic stimulation.

PPI analyses revealed that 376 of the 1119 genes could constitute an interconnected PPI network (Figure 7A). Furthermore, a network of 681 edges was identified, which was significantly greater than the expected 506 edges (*p* = 9.93 × 10^−14^). The genes in the top 10% of the 376 genes according to degree were defined as hub genes (Table SI 6). In addition, we depicted the spatiotemporal expression trajectories of the three hub genes with the highest degree values (i.e., cell division cycle 42, *CDC42*; mitogen‐activated protein kinase 3, *MAPK3;* and protein kinase C beta, *PRKCB*) (Figure 7B).

## 4 DISCUSSION

Using brain spatial information about the neuroplasticity of neural networks via left angular gyrus‐navigated rTMS and combining this information with regional gene expression profiles from AHBA transcriptomic data, possible mechanisms for improving cognitive dysfunction after rTMS intervention in AD‐spectrum patients with different sleep statuses were identified. Consistent with our hypothesis, this study revealed that multiple trajectories change angular gyrus anterior–posterior networks associated with the process of episodic memory improvement, and opposite network action patterns were exhibited in the two sleep quality groups. We also demonstrated that the interactions between sleep status and rTMS intervention significantly upregulated a highly interconnected number of genes in an interaction network, particularly those involved in a neurodegenerative pathway.

### 4.1 rTMS improves cognitive dysfunction by regulating angular gyrus networks in AD‐spectrum patients with different sleep statuses

Increasing evidence has indicated that left angular cortex navigation can significantly improve memory performance in healthy individuals; this improvement is attributed to the modulation of synaptic plasticity.^42^ Our previous studies also confirmed that via the targeted intervention of the left angular gyrus, cognitive dysfunction could be improved via dynamic regulation of the intra‐ and inter‐DMN^14^ and the neuroplasticity of the thalamic white matter system^13^ in AD‐spectrum patients. Considering the comprehensive effects of sleep status and rTMS intervention, the present study provides further evidence for the possibility of using left angular gyrus‐navigated rTMS protocols to improve cognitive impairments by selectively engaging and manipulating angular anterior–posterior networks in AD‐spectrum patients with different sleep statuses. However, this study revealed opposite network action patterns in the two sleep quality groups.

The physiological mechanism of sleep is associated with the strength and malleability of the thalamocortical circuits that underlie individual cognitive profiles.^43^ Sleep disorders can affect a particular cognitive process through the magnitude of global decline in general alertness and attention, specific cognition associated with emotion‐processing networks, and compensatory support in cortical regions.^44^ Moreover, accumulating evidence has shown that sleep disturbance contributes to cognitive decline,^45^ and sleep disturbance has increasingly been considered an inherent component of the degenerative process.^46^ Recent reviews have also highlighted advances in understanding the underlying relationship between sleep disorders and AD pathogenesis, suggesting that sleep is a potent modulator of Aβ deposition and pathological progression.^5^ Taken together, the present findings have implications for our understanding of left angular gyrus‐targeted rTMS and sleep disorders and the simultaneous modulation of brain anterior–posterior networks, facilitating the improvement of episodic memory dysfunctions.

### 4.2 Distinct variable neuroplasticity in angular gyrus anterior and posterior networks

The angular gyrus forms part of a wider lateral parietal cortex system with a core underlying neurocomputational function^47^ and a cross‐modal integrative hub.^48^, ^49^ In particular, in terms of the underlying architecture, anatomical evidence suggests that the angular gyrus is involved in episodic memory^48^ because it is a fundamental node within the DMN.^50^ Furthermore, the angular gyrus subregions were found to have varying underlying connectivity profiles, as the dorsal and ventral association fibers showed long‐range connectivity with lateral frontal executive control regions, the U‐shaped fibers showed connectivity with temporal lobe areas, and some additional projection fibers showed connectivity to the subcortical region and brainstem.^10^, ^47^, ^51^

However, these angular gyrus networks showed distinct variability in longitudinal changes in brain network differences between pre‐ and post‐rTMS treatment; linearly upward connectivity in the anterior network and linearly downward connectivity in the posterior network increased in AD‐spectrum patients with low sleep quality. Consistent with the findings of a recent study, the highly connected regions (i.e., angular gyrus) can exhibit both hypoconnectivity and hyperconnectivity, suggesting that the consequences of these compensatory processes themselves are also constrained by a certain balance, especially in brain hubs.^52^ Although the exact underlying mechanism is still unclear, two alternative interpretations are thought to be associated with these findings. First, this finding is a comprehensive result of high‐order interactions. Sleep disruption plays an important role in increasing Aβ deposition,^4^, ^53^ which occurs concurrently with the progressive disconnection of functional and anatomical network changes in preclinical subjects.^54^ However, there is a difference in the sequence of Aβ deposition caused by neurodegeneration and in the compensatory ability of the anterior and posterior brain regions.^55^ Second, angular anterior–posterior network variability may be associated with the segregation–integration balance theory of brain networks^56^ to support the heterogeneous demands of diverse cognitive abilities across individuals with different sleep disorders.

### 4.3 Interaction effect between sleep status and rTMS combined with genetic pathway information

The relationship between sleep status and gene expression in AD patients is complex. Sleep deprivation can disrupt normal gene expression patterns in the brain, leading to the accumulation of toxic proteins and the loss of neurons. rTMS has been found to improve sleep quality and duration, which can lead to an increase in neural plasticity and neurogenesis.^32^ By combining functional connectivity patterns in the interaction regions between sleep status and rTMS intervention with brain spatial gene expression data (i.e., 1119 genes) from cognitively impaired patients, we suggest that changes in angular networks occur through complex polygenetic and multipathway mechanisms in neurons and immune cells. These findings are consistent with previous evidence indicating that complex gene expression profiles are involved in the development of AD.^57^, ^58^, ^59^, ^60^ Specifically, this study revealed that these 1119 genes were functionally enriched in various molecular functions, cellular components, and biological processes involved in cognitive functions and sleep quality. Although the underlying mechanisms are still unclear, some potential explanations are as follows. A widely held belief is that the information‐processing capacity of the nervous system is largely dependent on the ability of neurons to communicate with each other; communication between neurons transmits information mainly through synapses.^61^, ^62^ Ion channels are critical for the generation of membrane potentials, neuronal growth and differentiation, signal transduction, and neurotransmitter release. The functional enrichment analyses in this study revealed that these genes were predominantly enriched in ion channels (ion‐gated channel activity) and synapses (synaptic membranes, synaptic assembly, and GABAergic synapses), suggesting that neuronal damage and gated channel abnormalities might be the mechanism of AD development.^63^, ^64^, ^65^ The brain networks are calculated on the basis of blood oxygen level‐dependent (BOLD) signaling; therefore, the association of changes in functional connectivity with the expression profiles of synaptic, ion‐channel‐enriched gene modules is compatible with the theory of neurovascular coupling of BOLD signaling, as BOLD signals can respond to synaptic activity.^66^, ^67^, ^68^ Therefore, AD‐enriched gene‐related regions (i.e., angular networks) may be targets of common attacks by risk factors in neurodegenerative diseases. In addition, multiple pathways participate in neurogenesis in individuals with cognitive impairment and related neurodegenerative diseases; for instance, Aβ could potently impair long‐term potentiation in the hippocampus through the cGMP‐PKG signaling pathway and cAMP signaling pathway^69^ and disrupt Ca^2+^ signaling in the calcium signaling pathway, mediating learning and memory disorders.^70^

Another way that rTMS may modulate the mRNA expression levels of target genes is through its effects on immune mechanisms in AD,^71^, ^72^ which can lead to a decrease in the production of inflammatory markers and a reduction in the severity of inflammation‐related symptoms. In this study, these screened genes were used to construct PPI networks supported by hub genes, which have functional implications for understanding AD pathology and treating cognitive disorders. Several related genes were identified by hub gene testing, suggesting that this approach may be utilized to explore the neurodegenerative and immune mechanisms of AD. For example, MAPK3 strongly influences the pathogenesis of AD through neuronal apoptosis, β‐secretase activity, and γ‐secretase activity.^73^ CDC42 serves as a critical modifier of neural morphology by enhancing neurite outgrowth and cone protrusion and could be highly related to cognitive impairment progression in AD via the regulation of T helper (Th) cells.^74^ PRKCB is associated with immune cell infiltration, and increased PRKCB expression promotes increases in the numbers of naive B cells, M1 macrophages, and other immune cells, while decreased PRKCB expression leads to severe immunodeficiency and memory impairment.^75^ Therefore, this modulation of mRNA expression may be one of the mechanisms by which rTMS is effective in treating AD.

Several limitations of this study should be noted. First, the MRI data and the gene expression data were not taken from the same individuals, which could contribute to the omission of genes of interest with large interindividual expression differences in the transcriptional–neuroimaging correlation analyses. Second, the gene expression profiles were age‐, sex‐, and race‐dependent, and age‐, sex‐, and race‐related mismatches between the imaging data may have introduced bias into the present study. Finally, all participants in this study were from a single center, and additional data from multiple centers are needed to validate our findings.

To our knowledge, this was the first transcription‐neuroimaging study to demonstrate that multitrajectory neuroplasticity in neural circuits was provoked via neuronavigated rTMS, which may also be associated with complex gene expression in AD‐spectrum patients with sleep disorders. These genes are associated with biological functions and disease pathways, suggesting that cognitive impairment and sleep disorders may occur through complex multigene and multipathway mechanisms and may serve as candidate genes for exploring the molecular mechanisms of neurodegeneration. The present strategy for integrating neuroimaging, rTMS intervention, and genetic data provides a new approach to comprehending the biological mechanisms involved in AD.

## CONFLICT OF INTEREST STATEMENT

The authors have no conflicts of interest to disclose. Author disclosures are available in the Supporting Information.

## CONSENT STATEMENT

This study was approved by the Ethics Committees of the Nanjing Drum Tower Hospital of Nanjing University Medical School. All participants provided informed consent before the experiment.

## Supporting information



Supporting information

Supporting information
