# Supplementary material for: Associations between the multitrajectory neuroplasticity of neuronavigated rTMS‐mediated angular gyrus networks and brain gene expression in AD spectrum patients with sleep disorders
Source: Alzheimers Dement. 2024 Sep 26;20(11):7885–901. doi: 10.1002/alz.14255 (PMC11567849; doi:10.1002/alz.14255)
Supplement: Supplementary file 1 — Supporting information [file ALZ-20-7885-s002.doc]

**Supporting Information**

**Associations between the multitrajectory neuroplasticity of neur-navigated rTMS-mediated angular networks and brain mRNA expression in AD-spectrum patients with sleep disorders**

Weina Yao^1,2,3^, Xinle Hou^2^, Huijuan Zhou^3^, Shengqi You^3^, Tingyu Lv^1,2,3^, Haifeng Chen^2,3^, Zhiyuan Yang^2^, Chang Chen^4^, Feng Bai ^2,5,6*^

1. Department of Neurology, Zhongnan Hospital of Wuhan University, Wuhan 430062, China

2. Department of Neurology, Nanjing Drum Tower Hospital, Affiliated Hospital of Medical School, Nanjing University, Nanjing 210008, China

3. Department of Neurology, Nanjing Drum Tower Hospital Clinical College of Traditional Chinese and Western Medicine, Nanjing University of Chinese Medicine, Nanjing 210008, China.

4. School of Elderly Care Services and Management, Nanjing University of Chinese Medicine, Nanjing 210023, China

5. Geriatric Medicine Center, Taikang Xianlin Drum Tower Hospital, Affiliated Hospital of Medical School, Nanjing University, Nanjing 210023, China.

6. Institute of Geriatric Medicine, Medical School of Nanjing University, Nanjing 210008, China

* Corresponding author

Feng Bai, E-mail address: baifeng515@126.com; baifeng@njglyy.com

**Figure SI 1.** After four weeks of neuro-navigation rTMS treatment, the CI+HSQ group exhibited significant improvements in neuropsychological performance. Thresholds were set at a corrected *p* < 0.05.


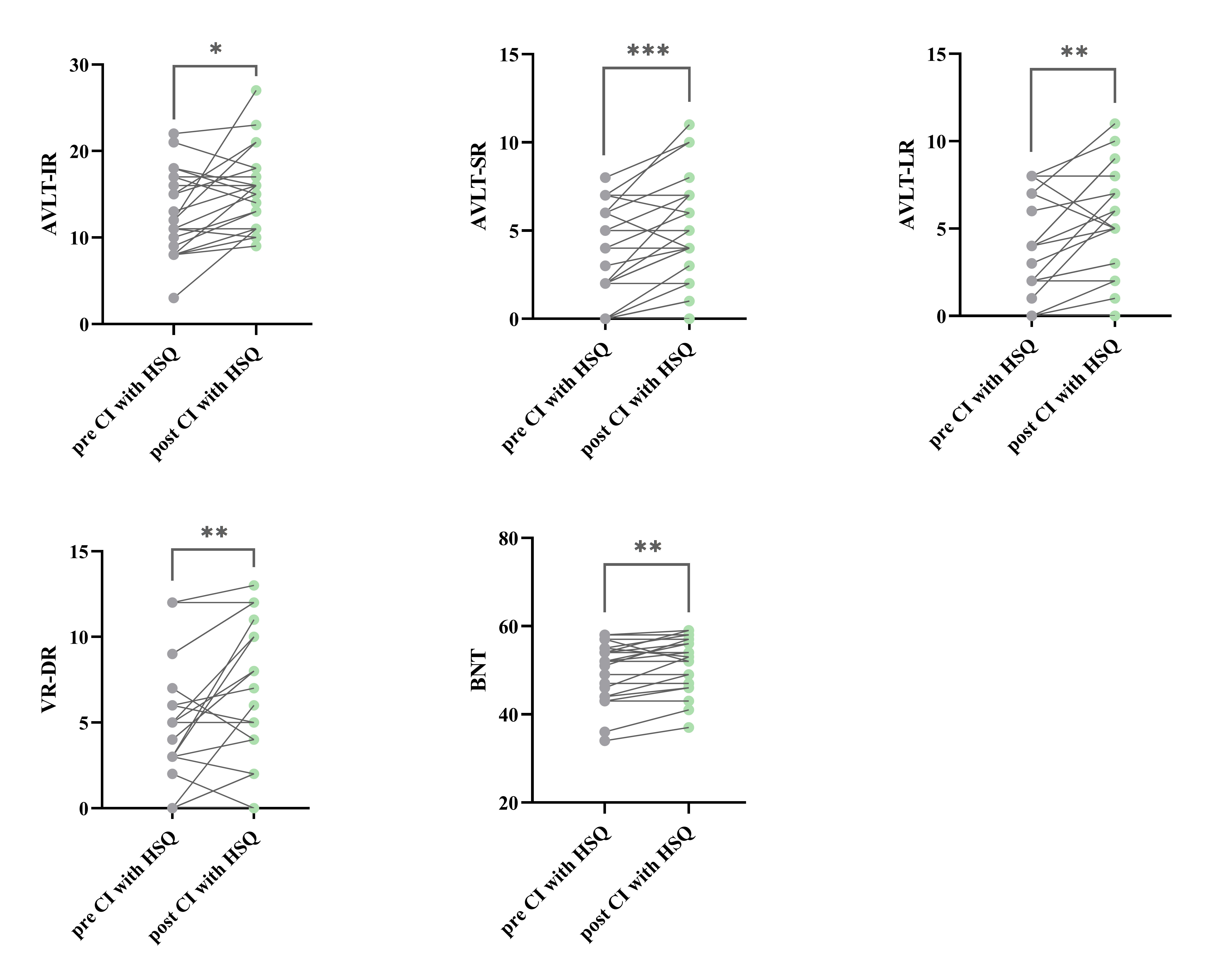


**Figure SI 2.** Specific expression of the genes associated with sleep status according to the rTMS intervention and ANOVA in the four groups. (a) Tissue-specific expression. (b) Cell-specific expression. (c-h) Temporal-specific expression. NPC, neural progenitor cell; OPC: oligodendrocyte progenitor cell.


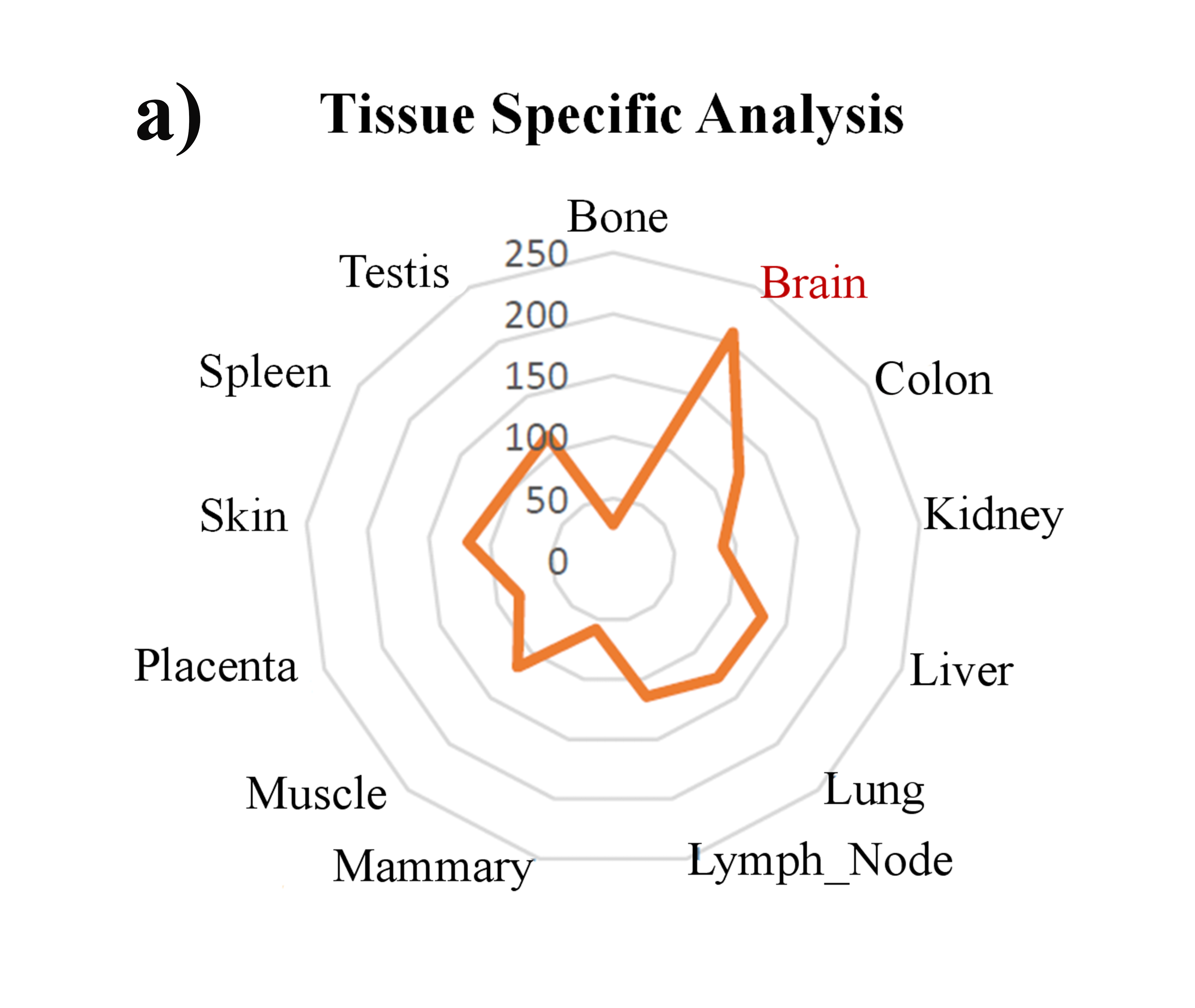


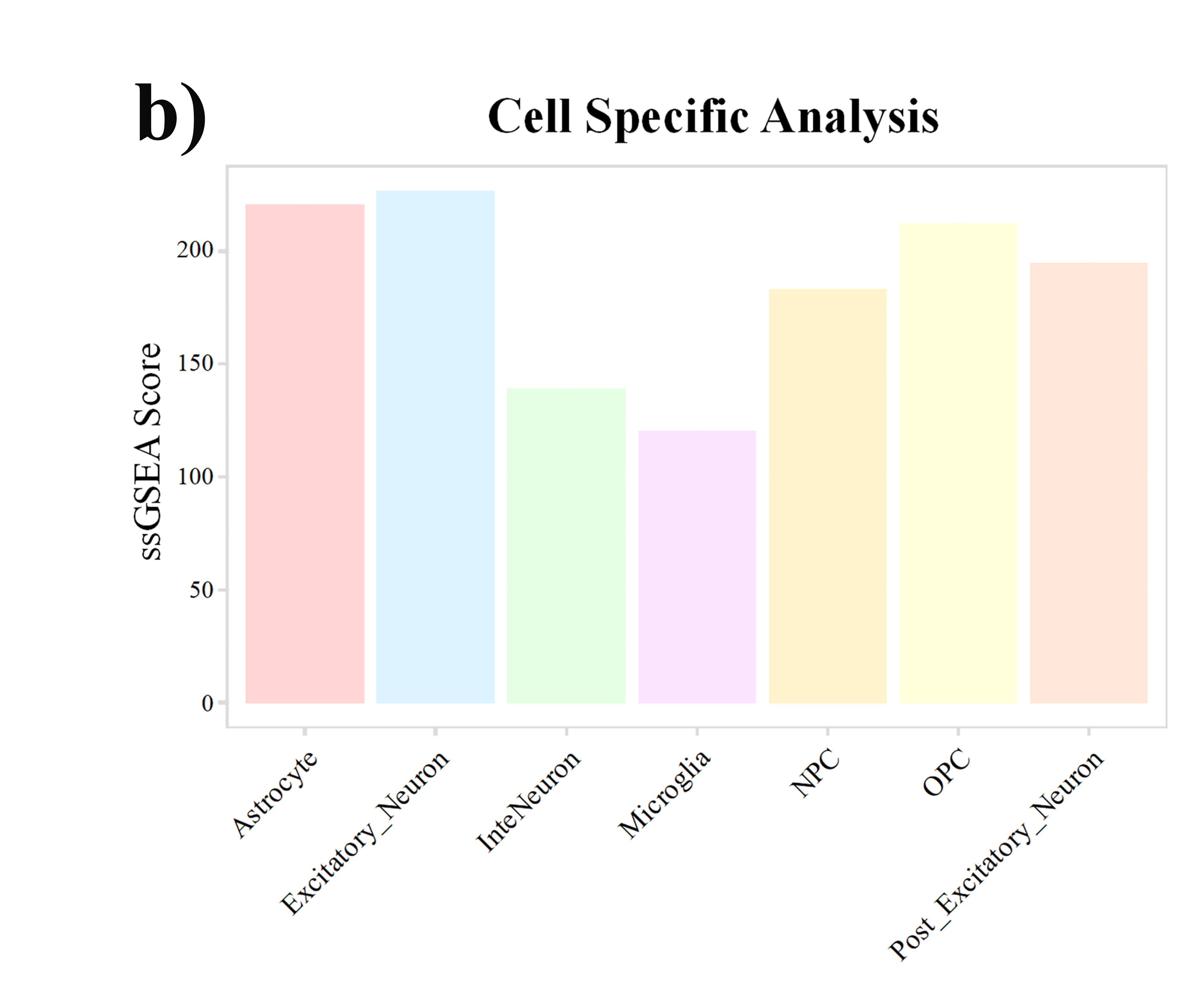

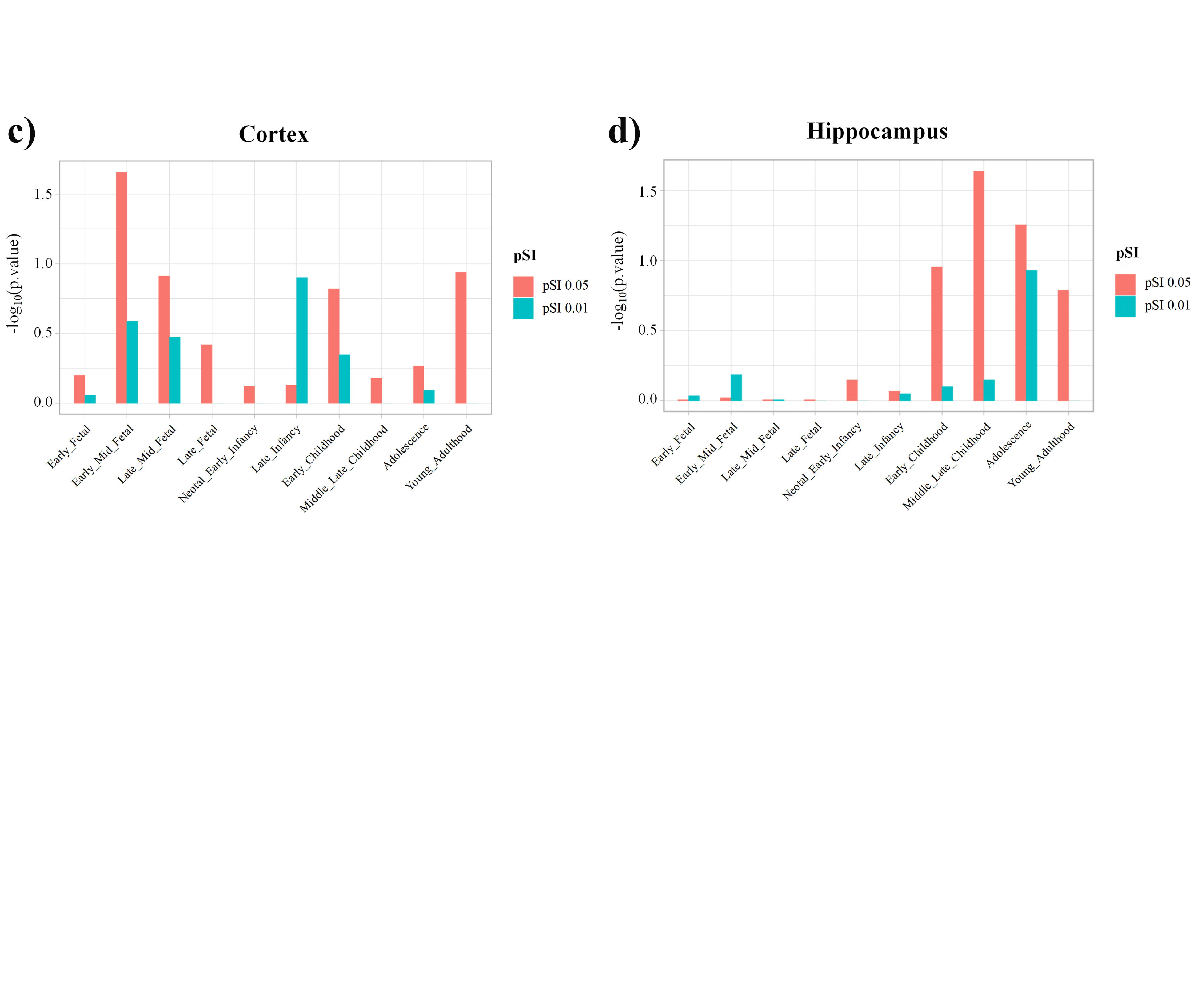

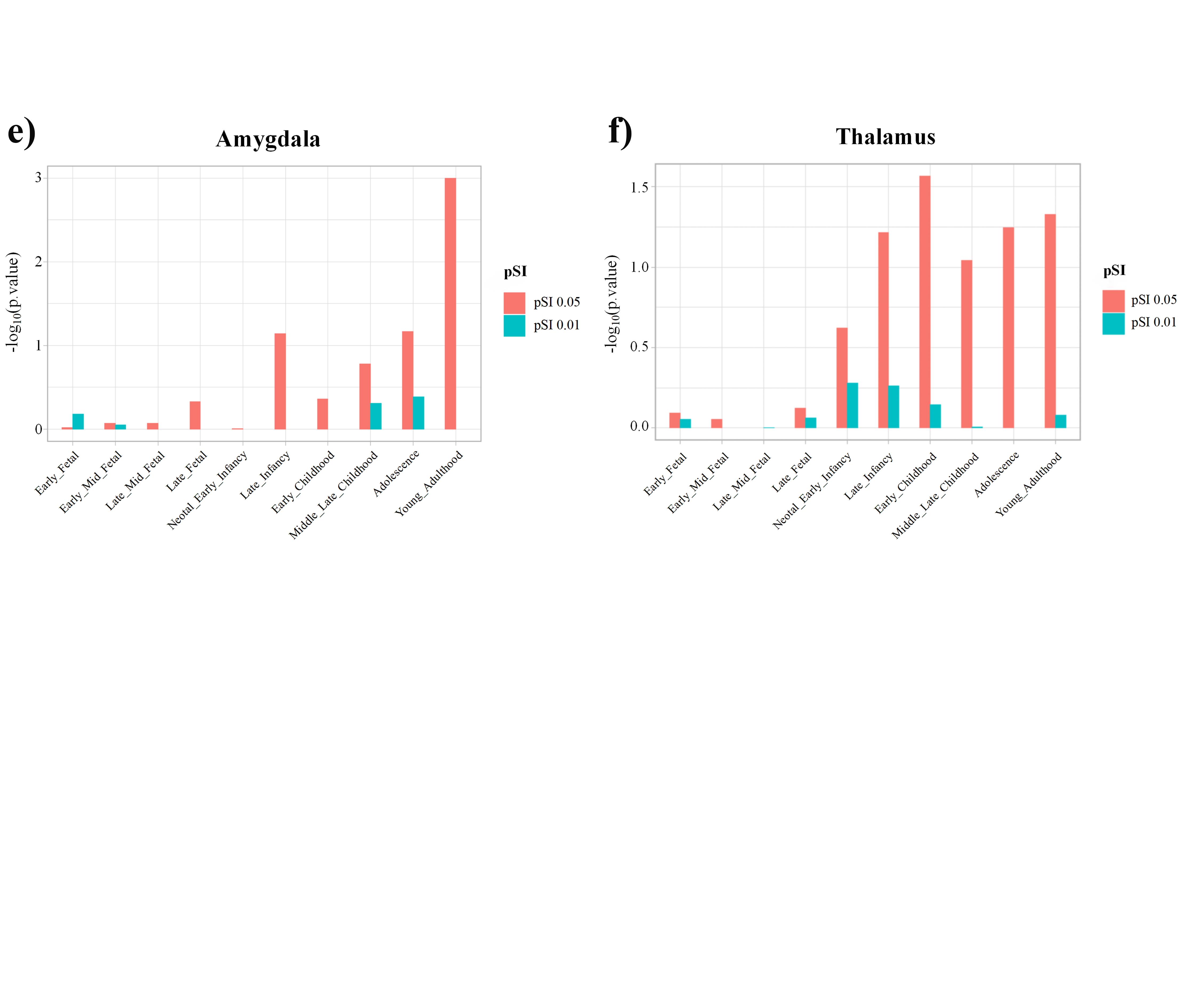

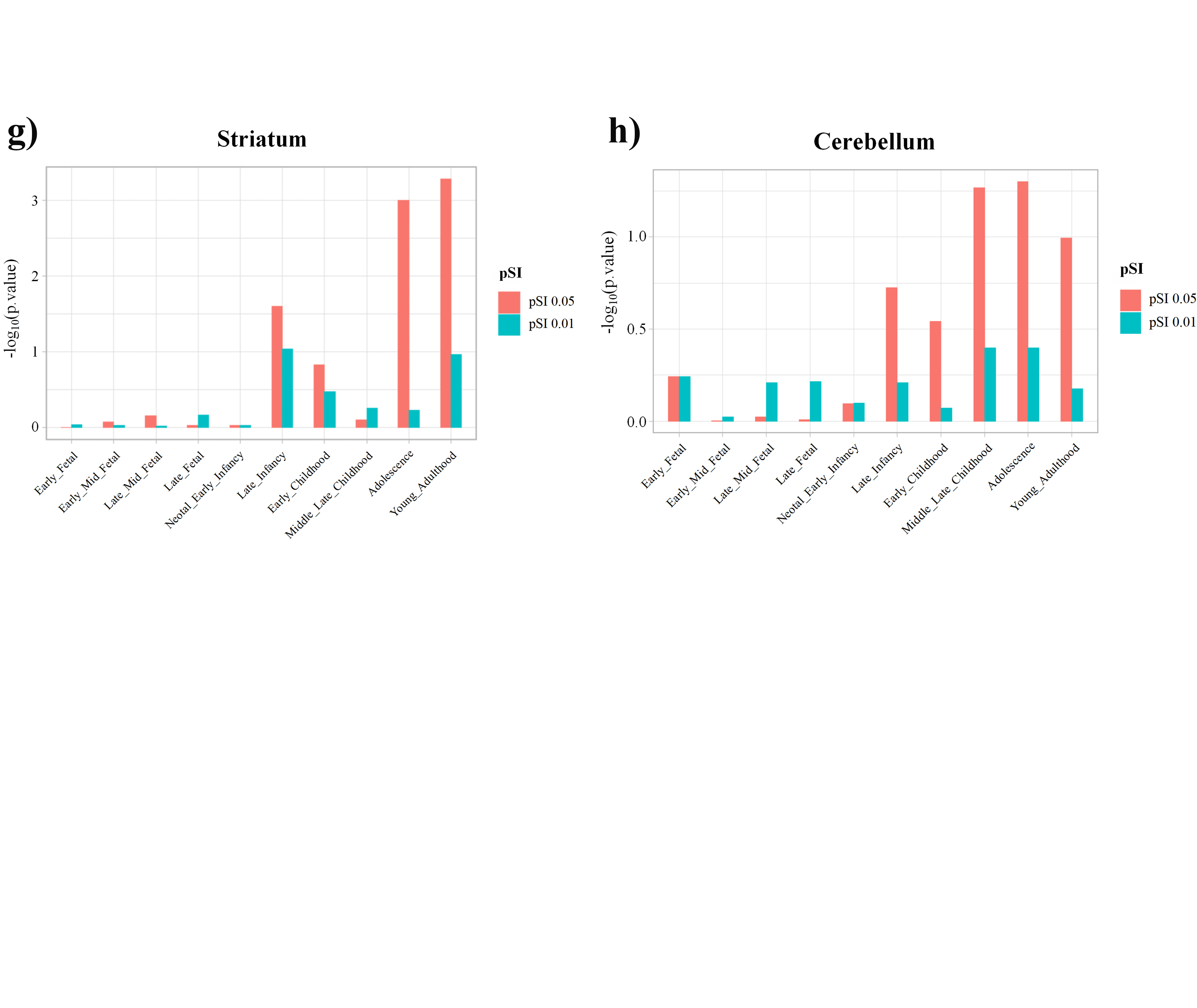


**Table SI 1. Correlation analysis associated with rTMS treatment and changes in neuropsychological tests and sleep status**

|  | **Items** |  | **ΔGeneral cognition** | | **ΔEpisodic**  **memory** | | | | **ΔProcessing**  **speed** | | | **ΔExecutive**  **Function** | | **ΔLanguage function** |
| --- | --- | --- | --- | --- | --- | --- | --- | --- | --- | --- | --- | --- | --- | --- |
|  |  |  | **MMSE** | **MoCA** | **AVLT-IR** | **AVLT-SR** | **AVLT-LR** | **VR-DR** | **TMT-A** | **StroopA** | **StroopB** | **StroopC** | **TMT-B** | **BNT** |
| **CI+**  **LSQ** | **ΔIS** | ***r*** | 0.184 | 0.033 | 0.167 | 0.114 | 0.136 | 0.110 | -0.159 | -0.079 | -0.178 | -0.395 | -0.224 | 0.272 |
|  |  | ***p*** | 0.401 | 0.882 | 0.446 | 0.605 | 0.537 | 0.618 | 0.468 | 0.721 | 0.416 | 0.062 | 0.303 | 0.209 |
|  | **ΔAIS** | ***r*** | -0.008 | -0.055 | 0.369 | 0.141 | 0.132 | 0.146 | -0.146 | -0.067 | -0.135 | -0.461 | -0.225 | 0.167 |
|  |  | ***p*** | 0.969 | 0.804 | 0.083 | 0.522 | 0.550 | 0.506 | 0.506 | 0.760 | 0.540 | 0.027* | 0.301 | 0.446 |
|  | **ΔPSQI** | ***r*** | 0.114 | -0.042 | 0.216 | 0.203 | 0.211 | 0.243 | -0.130 | -0.067 | -0.083 | -0.298 | -0.247 | 0.247 |
|  |  | ***p*** | 0.603 | 0.848 | 0.321 | 0.352 | 0.333 | 0.263 | 0.554 | 0.762 | 0.708 | 0.167 | 0.255 | 0.255 |
|  | **ΔSRSS** | ***r*** | -0.032 | 0.055 | 0.336 | 0.315 | 0.314 | 0.246 | -0.101 | -0.244 | -0.141 | -0.419 | -0.289 | 0.235 |
|  |  | ***p*** | 0.884 | 0.802 | 0.117 | 0.144 | 0.145 | 0.259 | 0.646 | 0.262 | 0.522 | 0.047* | 0.181 | 0.280 |
|  | **ΔESS** | ***r*** | -0.254 | -0.103 | 0.143 | 0.075 | -0.06 | 0.016 | -0.06 | -0.123 | -0.069 | -0.416 | -0.11 | 0.03 |
|  |  | ***p*** | 0.243 | 0.639 | 0.516 | 0.734 | 0.785 | 0.941 | 0.785 | 0.577 | 0.753 | 0.048* | 0.618 | 0.893 |
| **CI+**  **HSQ** | **ΔIS** | ***r*** | -0.102 | 0.042 | 0.073 | 0.445 | -0.232 | 0.262 | 0.02 | -0.295 | -0.382 | -0.357 | 0.03 | 0.201 |
|  |  | ***p*** | 0.642 | 0.849 | 0.741 | 0.033* | 0.287 | 0.226 | 0.927 | 0.172 | 0.072 | 0.095 | 0.894 | 0.357 |
|  | **ΔAIS** | ***r*** | -0.102 | 0.042 | 0.073 | 0.445 | -0.232 | 0.262 | 0.02 | -0.295 | -0.382 | -0.357 | 0.03 | 0.201 |
|  |  | ***p*** | 0.642 | 0.849 | 0.741 | 0.033* | 0.287 | 0.226 | 0.927 | 0.172 | 0.072 | 0.095 | 0.894 | 0.357 |
|  | **ΔPSQI** | ***r*** | -0.135 | 0.058 | 0.108 | 0.334 | -0.324 | 0.131 | 0.033 | -0.15 | -0.168 | -0.129 | 0.039 | 0.184 |
|  |  | ***p*** | 0.538 | 0.792 | 0.622 | 0.119 | 0.131 | 0.551 | 0.880 | 0.495 | 0.443 | 0.557 | 0.861 | 0.402 |
|  | **ΔSRSS** | ***r*** | 0.067 | 0.038 | 0.081 | 0.467 | -0.206 | 0.226 | 0.08 | -0.301 | -0.399 | -0.504 | 0.106 | 0.186 |
|  |  | ***p*** | 0.76 | 0.864 | 0.714 | 0.025* | 0.346 | 0.299 | 0.718 | 0.163 | 0.059 | 0.014* | 0.630 | 0.396 |
|  | **ΔESS** | ***r*** | 0.003 | -0.006 | -0.024 | 0.433 | 0.039 | 0.373 | -0.012 | -0.414 | -0.582 | -0.593 | 0.001 | 0.139 |
|  |  | ***p*** | 0.988 | 0.978 | 0.915 | 0.039* | 0.86 | 0.08 | 0.956 | 0.049* | 0.004** | 0.003** | 0.999 | 0.527 |

**Notes:** p values were obtained by Pearson's correlation between improvements in sleep quality and improvements in cognitive dysfunction after rTMS treatment. r represents the correlation coefficient. * indicates a significant difference between groups; * indicates *p* < 0.05. ** indicates *p* < 0.01.

**Abbreviations:** CI+LSQ, Cognitive Impairment Patients with Low Sleep Quality; CI+HSQ, Cognitive Impairment Patients with High Sleep Quality; MMSE, Mini Mental State Examination; MoCA, Montreal Cognitive Assessment; AVLT, Auditory Verbal Learning Test; AVLTH-SR, AVLTH-short recall; AVLTH-LR, AVLTH-long recall (3–5 min *vs*. 20 min delay); AVLTH-IR, AVLTH-immediate recall; VR-DR, Wechsler Memory Scale, visual reproduction delayed recall; StroopA, B and C, Stroop Color and Word Tests A, B and C; TMT-A and -B, Trail Making Tests-A and -B; BNT, Boston Naming Test; ISI, Insomnia Severity Index; AIS, Athens Insomnia Scale; PSQI, Pittsburgh Sleep Quality Index; SRSS, Self-Rating Scale of Sleep; ESS, Epworth Sleepiness Scale.

**Table SI 2. Sleep status × rTMS intervention**

| **Brain region** | **BA** | **Peak MNI**  **Coordiates x, y, z (mm)** | **Peak**  **F value** | **Cluster**  **size** |
| --- | --- | --- | --- | --- |
| *L Superior dorsal Frontal Gyrus* | 45 | -12, 21, 51 | 14.38 | 19575 |
| *L Superior Orbital Frontal Gyrus* | 47 | -21, 63, -3 | 13.02 | 17415 |
| *R Inferior Temporal Gyrus* | 37 | 45, -51, -18 | 8.48 | 11853 |
| *R SupraMarginal Gyrus* | 22 | 57, -36, 24 | 9.51 | 12933 |

**Note:** A corrected threshold by Monte Carlo simulation at *p* < 0.05. BA: Brodmann area; L= left; R= right; cluster size is in mm^3^; MNI: Montreal Neurological Institute.

**Table SI 3. Comparisons of connectivity between and within groups**

| **Items** | **CI+LSQ** | | | **CI+HSQ** | | | ***p* (CI+LSQ *vs.* CI+HSQ)** | |
| --- | --- | --- | --- | --- | --- | --- | --- | --- |
|  | **Pre**  **(n = 23)** | **Post**  **(n = 23)** | ***p*-value** | **Post**  **(n = 23)** | **Post**  **(n = 23)** | ***p*-value** | **Pre**  **(n = 23)** | **Post**  **(n = 23)** |
| **Connectivity at pre and post** | |  |  |  |  |  |  |  |
| *L Superior Frontal Gyrus* | 0.13 ± 0.15 | 0.23 ± 0.13 | 0.007** | 0.23 ± 0.16 | 0.11 ± 0.14 | 0.009** | 0.038* | 0.003** |
| *L Superior Orbital Frontal Gyrus* | 0.11 ± 0.13 | 0.24 ± 0.13 | 0.004** | 0.26 ± 0.13 | 0.15 ± 0.11 | 0.004** | <0.001*** | 0.026* |
| *R Inferior Temporal Gyrus* | -0.07 ± 0.11 | -0.17 ± 0.19 | 0.003** | -0.21 ± 0.15 | -0.10 ± 0.13 | 0.004** | <0.001*** | 0.136 |
| *R SupraMarginal Gyrus* | 0.01 ± 0.14 | -0.07 ± 0.17 | 0.005** | -0.09 ± 0.11 | 0.04 ± 0.13 | 0.002** | 0.005** | 0.005** |

**Notes:** Values are presented as the average ± standard deviation (SD). A paired samples Wilcoxon signed rank test was applied for each comparison. * indicates *p* < 0.05, ** indicates *p* < 0.01. *** indicates *p* < 0.001. L = left; R = right.

**Table SI 4. The genes associated with sleep status × rTMS intervention ANOVA in the four groups**

| **ID** | **Gene symbol** |
| --- | --- |
| 65 | ABHD12B |
| 88 | ABLIM3 |
| 93 | ABTB1 |
| 111 | ACAT2 |
| 119 | ACE |
| 165 | ACTC1 |
| 167 | ACTG1P4 |
| 176 | ACTR10 |
| 190 | ACVR1C |
| 191 | ACVR2A |
| 210 | ADAM28 |
| 214 | ADAM8 |
| 218 | ADAMTS13 |
| 232 | ADAP2 |
| 243 | ADCY1 |
| 246 | ADCY3 |
| 254 | ADCYAP1R1 |
| 270 | ADGRL2 |
| 271 | ADGRL3 |
| 299 | ADRB1 |
| 307 | AEBP2 |
| 315 | AFF2 |
| 341 | AGO1 |
| 346 | AGPAT3 |
| 416 | AKR7L |
| 430 | ALDH1A3 |
| 496 | AMH |
| 500 | AMMECR1 |
| 518 | ANAPC13 |
| 541 | ANKH |
| 560 | ANKRD18DP |
| 565 | ANKRD20A4-ANKRD20A20P |
| 567 | ANKRD20A8P |
| 570 | ANKRD24 |
| 578 | ANKRD33B |
| 586 | ANKRD37 |
| 591 | ANKRD45 |
| 594 | ANKRD50 |
| 598 | ANKRD6 |
| 601 | ANKS1A |
| 605 | ANKZF1 |
| 645 | AP1S3 |
| 648 | AP2B1 |
| 655 | AP3M2 |
| 670 | APBB1IP |
| 685 | APLN |
| 686 | APLNR |
| 691 | APOC1 |
| 724 | ARAP2 |
| 726 | ARC |
| 739 | ARFGEF3 |
| 746 | ARHGAP10 |
| 753 | ARHGAP22 |
| 757 | ARHGAP26 |
| 768 | ARHGAP36 |
| 770 | ARHGAP4 |
| 776 | ARHGAP6 |
| 789 | ARHGEF18 |
| 800 | ARHGEF40 |
| 812 | ARID5B |
| 851 | ARMCX4 |
| 857 | ARNTL |
| 884 | ART3 |
| 904 | ASB2 |
| 918 | ASGR2 |
| 934 | ASPDH |
| 948 | ASXL3 |
| 974 | ATG16L1 |
| 988 | ATL2 |
| 995 | ATOX1 |
| 996 | ATP10A |
| 1004 | ATP13A3 |
| 1015 | ATP2B1 |
| 1016 | ATP2B2 |
| 1019 | ATP2B4 |
| 1022 | ATP4A |
| 1027 | ATP5IF1 |
| 1067 | ATP8A2 |
| 1080 | ATRNL1 |
| 1082 | ATXN1 |
| 1089 | ATXN7L1 |
| 1090 | ATXN7L2 |
| 1094 | AUNIP |
| 1118 | B3GALT1 |
| 1159 | BAG3 |
| 1185 | BBC3 |
| 1190 | BBS10 |
| 1220 | BCL2A1 |
| 1230 | BCL7A |
| 1274 | BHLHE40 |
| 1277 | BICC1 |
| 1284 | BID |
| 1303 | BLVRB |
| 1344 | BPHL |
| 1374 | BRPF1 |
| 1425 | C10orf105 |
| 1446 | C11orf87 |
| 1463 | C12orf75 |
| 1472 | C15orf40 |
| 1530 | C1RL |
| 1550 | C1orf226 |
| 1554 | C1orf50 |
| 1581 | C2CD3 |
| 1584 | C2CD4C |
| 1602 | C2orf80 |
| 1687 | CA10 |
| 1697 | CA7 |
| 1706 | CABP1 |
| 1710 | CABP7 |
| 1711 | CABYR |
| 1725 | CACNA2D3 |
| 1729 | CACNB4 |
| 1731 | CACNG2 |
| 1734 | CACNG6 |
| 1741 | CADM1 |
| 1746 | CADPS2 |
| 1748 | CALB2 |
| 1756 | CALHM6 |
| 1772 | CAMK2D |
| 1775 | CAMK2N2 |
| 1778 | CAMKK2 |
| 1793 | CAPG |
| 1796 | CAPN12 |
| 1811 | CAPZB |
| 1842 | CASP1 |
| 1866 | CAVIN3 |
| 1870 | CBFB |
| 1933 | CCDC152 |
| 1951 | CCDC189 |
| 1956 | CCDC28A |
| 1962 | CCDC39 |
| 1969 | CCDC58 |
| 1971 | CCDC6 |
| 1983 | CCDC78 |
| 1987 | CCDC84 |
| 2004 | CCEPR |
| 2007 | CCIN |
| 2017 | CCL27 |
| 2037 | CCNI |
| 2043 | CCNO |
| 2058 | CCSER1 |
| 2063 | CCT5 |
| 2086 | CD24 |
| 2098 | CD33 |
| 2119 | CD74 |
| 2123 | CD83 |
| 2126 | CD8A |
| 2129 | CD99 |
| 2147 | CDC40 |
| 2148 | CDC42 |
| 2153 | CDC42EP3 |
| 2156 | CDC42P3 |
| 2182 | CDH4 |
| 2219 | CDKL1 |
| 2227 | CDKN2A |
| 2251 | CEBPB |
| 2262 | CELF2 |
| 2269 | CEMIP |
| 2288 | CENPW |
| 2297 | CEP152 |
| 2304 | CEP192 |
| 2321 | CEP85L |
| 2323 | CEP95 |
| 2346 | CFAP46 |
| 2351 | CFAP69 |
| 2358 | CFD |
| 2365 | CFLAR |
| 2380 | CHAF1A |
| 2408 | CHGB |
| 2416 | CHML |
| 2428 | CHN2 |
| 2439 | CHRM1 |
| 2442 | CHRM4 |
| 2445 | CHRNA2 |
| 2478 | CIB2 |
| 2496 | CIT |
| 2507 | CKMT1B |
| 2510 | CKS1B |
| 2541 | CLEC16A |
| 2548 | CLEC4G |
| 2560 | CLIP1 |
| 2568 | CLK4 |
| 2570 | CLMP |
| 2600 | CMAHP |
| 2601 | CMAS |
| 2606 | CMIP |
| 2610 | CMSS1 |
| 2611 | CMTM3 |
| 2615 | CMTM7 |
| 2616 | CMTM8 |
| 2625 | CNGB1 |
| 2636 | CNNM1 |
| 2660 | CNST |
| 2688 | COG2 |
| 2700 | COL13A1 |
| 2718 | COL5A2 |
| 2726 | COL8A2 |
| 2785 | CORO6 |
| 2813 | CPAMD8 |
| 2820 | CPLX1 |
| 2830 | CPNE6 |
| 2833 | CPNE9 |
| 2879 | CRH |
| 2895 | CRNDE |
| 2960 | CSRNP3 |
| 2984 | CTDSPL |
| 2994 | CTNNAL1 |
| 3026 | CTXN3 |
| 3040 | CUX1 |
| 3078 | CYB5R1 |
| 3080 | CYB5R3 |
| 3101 | CYP26B1 |
| 3123 | CYR61 |
| 3124 | CYS1 |
| 3133 | CYTOR |
| 3144 | DACT1 |
| 3155 | DAPK1 |
| 3156 | DAPK1-IT1 |
| 3188 | DCAF4 |
| 3203 | DCLK1 |
| 3230 | DDA1 |
| 3269 | DDX46 |
| 3276 | DDX54 |
| 3296 | DEFB131A |
| 3299 | DEGS1 |
| 3307 | DENND2D |
| 3326 | DERL3 |
| 3345 | DGKG |
| 3353 | DHCR7 |
| 3355 | DHDH |
| 3365 | DHRS2 |
| 3401 | DIO3 |
| 3402 | DIO3OS |
| 3406 | DIRAS1 |
| 3408 | DIRAS3 |
| 3436 | DLGAP1-AS4 |
| 3443 | DLL3 |
| 3445 | DLX1 |
| 3446 | DLX2 |
| 3454 | DMKN |
| 3471 | DNAH14 |
| 3494 | DNAJC12 |
| 3512 | DNAJC4 |
| 3564 | DOK6 |
| 3591 | DPP7 |
| 3592 | DPP8 |
| 3598 | DPY19L2P1 |
| 3599 | DPY19L2P2 |
| 3601 | DPY19L2P4 |
| 3607 | DPYSL3 |
| 3617 | DRD1 |
| 3624 | DRP2 |
| 3640 | DTNBP1 |
| 3664 | DUSP2 |
| 3671 | DUSP5 |
| 3673 | DUSP6 |
| 3674 | DUSP7 |
| 3680 | DVL2 |
| 3686 | DYNC1I1 |
| 3725 | EBP |
| 3733 | ECHDC3 |
| 3769 | EEPD1 |
| 3791 | EFNA5 |
| 3805 | EGR1 |
| 3806 | EGR2 |
| 3807 | EGR3 |
| 3827 | EIF2A |
| 3899 | ELMOD2 |
| 3965 | ENO1 |
| 3968 | ENO3 |
| 3994 | EPB41 |
| 4001 | EPB41L4B |
| 4020 | EPHB6 |
| 4024 | EPHX4 |
| 4029 | EPN3 |
| 4090 | ESPL1 |
| 4095 | ESRRA |
| 4113 | ETV1 |
| 4115 | ETV5 |
| 4116 | ETV6 |
| 4158 | EXTL2 |
| 4169 | F12 |
| 4184 | FABP3 |
| 4185 | FABP5 |
| 4186 | FABP5P3 |
| 4188 | FABP7 |
| 4208 | FAM105A |
| 4235 | FAM129B |
| 4250 | FAM13C |
| 4284 | FAM181A |
| 4301 | FAM198B |
| 4317 | FAM20A |
| 4323 | FAM213A |
| 4329 | FAM217B |
| 4345 | FAM241B |
| 4361 | FAM49B |
| 4378 | FAM78A |
| 4380 | FAM81A |
| 4392 | FAM89A |
| 4398 | FAM92A |
| 4416 | FANCL |
| 4419 | FAP |
| 4421 | FAR2 |
| 4432 | FASTKD1 |
| 4437 | FAT3 |
| 4448 | FBLN7 |
| 4473 | FBXO2 |
| 4483 | FBXO32 |
| 4484 | FBXO33 |
| 4500 | FBXO9 |
| 4521 | FDFT1 |
| 4533 | FER1L4 |
| 4536 | FES |
| 4539 | FEZF2 |
| 4544 | FGD3 |
| 4555 | FGF18 |
| 4577 | FHL2 |
| 4581 | FHOD3 |
| 4587 | FIGN |
| 4596 | FIZ1 |
| 4607 | FKBP5 |
| 4623 | FLJ33534 |
| 4634 | FLRT2 |
| 4645 | FMN1 |
| 4646 | FMN2 |
| 4663 | FNDC4 |
| 4676 | FOSB |
| 4678 | FOSL2 |
| 4690 | FOXG1 |
| 4726 | FRMD3 |
| 4727 | FRMD4A |
| 4732 | FRMPD3 |
| 4748 | FSTL4 |
| 4749 | FSTL5 |
| 4767 | FUOM |
| 4781 | FXN |
| 4787 | FXYD6 |
| 4789 | FYB1 |
| 4824 | GABPB1-IT1 |
| 4826 | GABRA1 |
| 4828 | GABRA3 |
| 4829 | GABRA4 |
| 4831 | GABRB1 |
| 4832 | GABRB2 |
| 4834 | GABRD |
| 4872 | GALNTL5 |
| 4874 | GALR1 |
| 4900 | GASAL1 |
| 4931 | GCNT4 |
| 4950 | GDPD5 |
| 4962 | GFAP |
| 4999 | GHR |
| 5002 | GHRLOS |
| 5032 | GK |
| 5040 | GLCCI1 |
| 5059 | GLOD4 |
| 5061 | GLRA2 |
| 5074 | GLTPD2 |
| 5075 | GLUD1 |
| 5076 | GLUD2 |
| 5097 | GNA14 |
| 5120 | GNG4 |
| 5122 | GNG7 |
| 5161 | GON4L |
| 5191 | GPC4 |
| 5197 | GPD2 |
| 5232 | GPR158 |
| 5233 | GPR161 |
| 5259 | GPR63 |
| 5260 | GPR68 |
| 5269 | GPRIN1 |
| 5270 | GPRIN2 |
| 5304 | GRID2 |
| 5311 | GRIN2A |
| 5315 | GRIN3A |
| 5324 | GRK6 |
| 5331 | GRM8 |
| 5333 | GRP |
| 5342 | GSDMB |
| 5387 | GTF2IRD2 |
| 5407 | GUCY1A2 |
| 5421 | GYG2 |
| 5450 | HACL1 |
| 5463 | HAPLN4 |
| 5497 | HCLS1 |
| 5505 | HCST |
| 5570 | HES6 |
| 5675 | HIVEP2 |
| 5678 | HK2 |
| 5683 | HLA-DMA |
| 5685 | HLA-DOA |
| 5687 | HLA-DPA1 |
| 5689 | HLA-DPB2 |
| 5690 | HLA-DQB1 |
| 5693 | HLA-DRB1 |
| 5705 | HLF |
| 5764 | HNRNPUL2 |
| 5772 | HOPX |
| 5793 | HPS1 |
| 5799 | HR |
| 5805 | HRH2 |
| 5815 | HS3ST5 |
| 5858 | HSPB11 |
| 5873 | HTR1F |
| 5875 | HTR2C |
| 5905 | ICA1 |
| 5927 | IDI1 |
| 5930 | IDS |
| 5935 | IER5 |
| 5936 | IER5L |
| 5941 | IFI27L1 |
| 5942 | IFI27L2 |
| 5977 | IFT57 |
| 5983 | IGDCC4 |
| 5991 | IGFBP2 |
| 6038 | IL1B |
| 6039 | IL1RAP |
| 6074 | IMPACT |
| 6081 | INAFM1 |
| 6104 | INPP5A |
| 6126 | INTS4 |
| 6140 | IPCEF1 |
| 6178 | IRF5 |
| 6191 | ISG15 |
| 6199 | ISOC1 |
| 6218 | ITGA9 |
| 6228 | ITGB3BP |
| 6231 | ITGB7 |
| 6238 | ITPA |
| 6239 | ITPK1 |
| 6243 | ITPR1 |
| 6269 | JAM2 |
| 6276 | JDP2 |
| 6284 | JMJD7 |
| 6292 | JPT1 |
| 6303 | KALRN |
| 6330 | KBTBD12 |
| 6335 | KBTBD6 |
| 6337 | KBTBD8 |
| 6339 | KCNA1 |
| 6340 | KCNA2 |
| 6341 | KCNA3 |
| 6346 | KCNAB3 |
| 6347 | KCNB1 |
| 6356 | KCNE4 |
| 6358 | KCNF1 |
| 6359 | KCNG1 |
| 6361 | KCNH1 |
| 6362 | KCNH2 |
| 6364 | KCNH4 |
| 6371 | KCNIP3 |
| 6374 | KCNJ11 |
| 6376 | KCNJ14 |
| 6380 | KCNJ4 |
| 6395 | KCNMA1 |
| 6409 | KCNS1 |
| 6410 | KCNS2 |
| 6412 | KCNT1 |
| 6414 | KCNV1 |
| 6416 | KCTD1 |
| 6417 | KCTD10 |
| 6419 | KCTD12 |
| 6423 | KCTD16 |
| 6430 | KCTD4 |
| 6436 | KDELC1 |
| 6486 | KIAA1024 |
| 6487 | KIAA1107 |
| 6492 | KIAA1211 |
| 6505 | KIAA1614 |
| 6507 | KIAA1671 |
| 6519 | KIF17 |
| 6527 | KIF21B |
| 6534 | KIF2A |
| 6556 | KITLG |
| 6564 | KLF12 |
| 6576 | KLF9 |
| 6583 | KLHDC8A |
| 6586 | KLHL1 |
| 6600 | KLHL29 |
| 6614 | KLK10 |
| 6631 | KLRG1 |
| 6639 | KMT5B |
| 6642 | KNG1 |
| 6658 | KRBOX4 |
| 6660 | KREMEN1 |
| 6682 | KRT86 |
| 6739 | KYAT1 |
| 6744 | L2HGDH |
| 6758 | LAMA2 |
| 6771 | LAMP5 |
| 6796 | LAT |
| 6797 | LAT2 |
| 6818 | LCE3C |
| 6830 | LCP1 |
| 6835 | LDB2 |
| 6841 | LDHD |
| 6876 | LGR4 |
| 6896 | LIG1 |
| 6901 | LILRB4 |
| 6908 | LIMK2 |
| 6911 | LIN28B |
| 6917 | LIN7B |
| 6919 | LIN9 |
| 6926 | LINC00260 |
| 6928 | LINC00266-1 |
| 6940 | LINC00473 |
| 6946 | LINC00507 |
| 6950 | LINC00595 |
| 6952 | LINC00599 |
| 6963 | LINC00667 |
| 6969 | LINC00842 |
| 6972 | LINC00886 |
| 6973 | LINC00889 |
| 6995 | LINC01102 |
| 7000 | LINC01128 |
| 7006 | LINC01158 |
| 7014 | LINC01315 |
| 7053 | LINC02217 |
| 7055 | LINC02361 |
| 7056 | LINC02381 |
| 7061 | LINGO2 |
| 7085 | LMCD1 |
| 7091 | LMO1 |
| 7095 | LMO7 |
| 7118 | LOC100130331 |
| 7146 | LOC100505938 |
| 7169 | LOC101060391 |
| 7172 | LOC101927027 |
| 7184 | LOC101928087 |
| 7189 | LOC101928433 |
| 7203 | LOC102724156 |
| 7216 | LOC105376360 |
| 7285 | LOC642852 |
| 7305 | LOC728392 |
| 7319 | LOC729737 |
| 7327 | LOC93622 |
| 7336 | LOXL1 |
| 7342 | LPAR6 |
| 7404 | LRRC38 |
| 7405 | LRRC39 |
| 7406 | LRRC3B |
| 7410 | LRRC42 |
| 7422 | LRRC6 |
| 7437 | LRRFIP1 |
| 7441 | LRRK2 |
| 7444 | LRRN3 |
| 7463 | LSM4 |
| 7472 | LST1 |
| 7475 | LTB |
| 7481 | LTBP4 |
| 7484 | LTK |
| 7486 | LTV1 |
| 7492 | LUZP2 |
| 7493 | LXN |
| 7498 | LY6H |
| 7499 | LY86 |
| 7508 | LYPD1 |
| 7521 | LYRM9 |
| 7525 | LYSMD4 |
| 7533 | LZTS3 |
| 7536 | MAB21L2 |
| 7542 | MAD1L1 |
| 7553 | MAFB |
| 7561 | MAGED1 |
| 7585 | MAML1 |
| 7593 | MAN1B1-AS1 |
| 7603 | MANEAL |
| 7611 | MAP1LC3A |
| 7627 | MAP3K13 |
| 7628 | MAP3K14 |
| 7652 | MAPK10 |
| 7658 | MAPK3 |
| 7690 | MARCKS |
| 7706 | MAST4 |
| 7711 | MATN2 |
| 7735 | MBOAT7 |
| 7740 | MC1R |
| 7744 | MCCC1 |
| 7753 | MCHR2 |
| 7776 | MCUR1 |
| 7791 | ME2 |
| 7813 | MED21 |
| 7830 | MEF2A |
| 7843 | MEIKIN |
| 7848 | MELTF |
| 7854 | MESP1 |
| 7895 | MFGE8 |
| 7911 | MFSD4A |
| 7923 | MGAT4A |
| 7939 | MGST1 |
| 7948 | MICA |
| 7975 | MINCR |
| 7982 | MIOS |
| 7991 | MIR29B2CHG |
| 7992 | MIR4435-2HG |
| 7993 | MIR4697HG |
| 7995 | MIR600HG |
| 7998 | MIR9-3HG |
| 8013 | MKNK2 |
| 8029 | MLLT11 |
| 8040 | MMAB |
| 8063 | MOB2 |
| 8076 | MOK |
| 8100 | MPDZ |
| 8106 | MPHOSPH9 |
| 8114 | MPP3 |
| 8134 | MREG |
| 8140 | MRGPRF |
| 8153 | MROH8 |
| 8180 | MRPL37 |
| 8222 | MRPS30 |
| 8223 | MRPS30-DT |
| 8230 | MRPS6 |
| 8261 | MSRB3 |
| 8283 | MTBP |
| 8349 | MUM1 |
| 8397 | MYH7B |
| 8400 | MYL12B |
| 8420 | MYO1B |
| 8427 | MYO5B |
| 8430 | MYO7A |
| 8444 | MYRIP |
| 8475 | NABP1 |
| 8489 | NAGK |
| 8491 | NAGPA |
| 8499 | NANOS1 |
| 8511 | NAPEPLD |
| 8525 | NAT8L |
| 8560 | NCEH1 |
| 8581 | NCR2 |
| 8589 | NDN |
| 8625 | NDUFB1 |
| 8651 | NEB |
| 8657 | NECAP1 |
| 8665 | NEDD9 |
| 8670 | NEIL1 |
| 8685 | NELL1 |
| 8711 | NEXN |
| 8726 | NFIC |
| 8750 | NHLRC1 |
| 8762 | NIM1K |
| 8777 | NIPSNAP3A |
| 8781 | NIT2 |
| 8783 | NKAIN2 |
| 8796 | NKX1-2 |
| 8806 | NLE1 |
| 8890 | NPAS2 |
| 8893 | NPB |
| 8924 | NPTX1 |
| 8936 | NR1D1 |
| 8937 | NR1D2 |
| 8940 | NR2C1 |
| 8946 | NR2F2 |
| 8949 | NR3C1 |
| 8950 | NR3C2 |
| 8954 | NRAP |
| 8992 | NSFL1C |
| 9017 | NT5M |
| 9025 | NTN4 |
| 9028 | NTNG2 |
| 9030 | NTRK2 |
| 9033 | NTSR2 |
| 9048 | NUDT11 |
| 9059 | NUDT22 |
| 9071 | NUMBL |
| 9081 | NUP35 |
| 9093 | NUPR1 |
| 9094 | NUPR2 |
| 9096 | NUTF2 |
| 9114 | NYNRIN |
| 9153 | OLFM4 |
| 9163 | ONECUT1 |
| 9164 | ONECUT2 |
| 9171 | OPN3 |
| 9176 | OPTN |
| 9188 | OR14I1 |
| 9201 | OR2L8 |
| 9249 | OSBP2 |
| 9254 | OSBPL3 |
| 9266 | OST4 |
| 9287 | OTX1 |
| 9288 | OVGP1 |
| 9295 | OXNAD1 |
| 9300 | OXTR |
| 9305 | P2RX6 |
| 9316 | P3H3 |
| 9364 | PALMD |
| 9385 | PAQR3 |
| 9413 | PARP8 |
| 9417 | PART1 |
| 9420 | PASK |
| 9429 | PAX8 |
| 9449 | PCBD1 |
| 9465 | PCDH19 |
| 9471 | PCDHA11 |
| 9474 | PCDHB10 |
| 9475 | PCDHB11 |
| 9480 | PCDHB16 |
| 9481 | PCDHB17P |
| 9482 | PCDHB18P |
| 9488 | PCDHB8 |
| 9489 | PCDHB9 |
| 9500 | PCED1B |
| 9509 | PCK2 |
| 9526 | PCP4 |
| 9528 | PCSK1 |
| 9532 | PCSK5 |
| 9556 | PDE10A |
| 9562 | PDE2A |
| 9585 | PDGFRA |
| 9607 | PDP1 |
| 9629 | PEA15 |
| 9637 | PEG10 |
| 9648 | PER1 |
| 9649 | PER2 |
| 9656 | PEX10 |
| 9664 | PEX19 |
| 9678 | PFKFB2 |
| 9707 | PGM2L1 |
| 9711 | PGRMC1 |
| 9752 | PHLDB2 |
| 9784 | PID1 |
| 9831 | PIN4P1 |
| 9834 | PINLYP |
| 9849 | PIRT |
| 9851 | PITHD1 |
| 9862 | PJVK |
| 9868 | PKDCC |
| 9869 | PKIA |
| 9870 | PKIB |
| 9900 | PLAGL1 |
| 9901 | PLAGL2 |
| 9908 | PLCB1 |
| 9923 | PLCXD3 |
| 9930 | PLEKHA1 |
| 9935 | PLEKHA6 |
| 9956 | PLEKHM2 |
| 9982 | PLPP4 |
| 9987 | PLPPR2 |
| 9997 | PLXDC1 |
| 9998 | PLXDC2 |
| 10011 | PMEPA1 |
| 10032 | PNISR |
| 10035 | PNLDC1 |
| 10042 | PNMA8A |
| 10060 | POC1A |
| 10132 | PON3 |
| 10146 | POU3F1 |
| 10148 | POU3F3 |
| 10151 | POU6F1 |
| 10152 | POU6F2 |
| 10153 | PP12613 |
| 10158 | PPARD |
| 10160 | PPARGC1A |
| 10165 | PPCS |
| 10210 | PPP1CB |
| 10255 | PPP2R2D |
| 10276 | PPP5D1 |
| 10292 | PRAG1 |
| 10306 | PRDM2 |
| 10311 | PRDX1 |
| 10323 | PREP |
| 10351 | PRKCB |
| 10377 | PRMT2 |
| 10381 | PRMT7 |
| 10382 | PRMT8 |
| 10408 | PRPF4B |
| 10411 | PRPH |
| 10412 | PRPH2 |
| 10415 | PRPS2 |
| 10427 | PRR22 |
| 10429 | PRR29 |
| 10433 | PRR5 |
| 10445 | PRRT3 |
| 10449 | PRSS1 |
| 10450 | PRSS16 |
| 10451 | PRSS2 |
| 10458 | PRSS3P2 |
| 10488 | PSMB2 |
| 10493 | PSMB7 |
| 10504 | PSMD10 |
| 10507 | PSMD13 |
| 10543 | PTCHD1 |
| 10544 | PTCHD4 |
| 10553 | PTGER3 |
| 10554 | PTGER4 |
| 10556 | PTGES |
| 10560 | PTGIS |
| 10563 | PTGS2 |
| 10568 | PTK2B |
| 10591 | PTPN4 |
| 10594 | PTPRA |
| 10596 | PTPRC |
| 10599 | PTPRD-AS1 |
| 10610 | PTPRR |
| 10618 | PTS |
| 10629 | PURB |
| 10653 | PXYLP1 |
| 10654 | PYCARD |
| 10658 | PYDC1 |
| 10709 | RAB27B |
| 10721 | RAB35 |
| 10722 | RAB36 |
| 10723 | RAB37 |
| 10727 | RAB3B |
| 10737 | RAB40C |
| 10767 | RAC2 |
| 10783 | RAD54B |
| 10788 | RAE1 |
| 10789 | RAET1E-AS1 |
| 10840 | RARB |
| 10853 | RASAL3 |
| 10855 | RASD2 |
| 10862 | RASGRP2 |
| 10885 | RBBP4 |
| 10933 | RBMS1 |
| 10941 | RBP4 |
| 11022 | RFC3 |
| 11048 | RGL2 |
| 11051 | RGMB |
| 11079 | RHBDD2 |
| 11082 | RHBDF2 |
| 11117 | RILP |
| 11125 | RIMS3 |
| 11130 | RING1 |
| 11141 | RIPOR2 |
| 11159 | RMND5A |
| 11166 | RNASEH2A |
| 11170 | RNASET2 |
| 11199 | RNF149 |
| 11205 | RNF165 |
| 11230 | RNF220 |
| 11265 | ROCK2 |
| 11272 | RORB |
| 11289 | RPGR |
| 11291 | RPH3A |
| 11405 | RPS6KA3 |
| 11427 | RRAGB |
| 11465 | RSPO4 |
| 11471 | RTBDN |
| 11477 | RTKN2 |
| 11517 | RXFP1 |
| 11527 | S100A10 |
| 11563 | SAMD4A |
| 11567 | SAMD9 |
| 11590 | SAT2 |
| 11592 | SATB2 |
| 11611 | SCAI |
| 11629 | SCCPDH |
| 11646 | SCLT1 |
| 11650 | SCN1B |
| 11659 | SCN9A |
| 11667 | SCPEP1 |
| 11673 | SCRT1 |
| 11711 | SEC11C |
| 11733 | SEC61A2 |
| 11762 | SEMA3D |
| 11765 | SEMA4A |
| 11769 | SEMA4F |
| 11811 | SERPINA1 |
| 11817 | SERPINB8 |
| 11829 | SERTAD4 |
| 11837 | SETBP1 |
| 11847 | SETDB1 |
| 11888 | SGIP1 |
| 11898 | SGSH |
| 11899 | SGSM1 |
| 11922 | SH3GL1 |
| 11972 | SIGIRR |
| 11985 | SIN3B |
| 12005 | SKAP2 |
| 12007 | SKIDA1 |
| 12013 | SLA |
| 12029 | SLC12A8 |
| 12035 | SLC15A3 |
| 12046 | SLC16A8 |
| 12062 | SLC1A4 |
| 12084 | SLC24A2 |
| 12114 | SLC25A37 |
| 12135 | SLC26A10 |
| 12136 | SLC26A11 |
| 12151 | SLC29A4 |
| 12164 | SLC30A10 |
| 12166 | SLC30A4 |
| 12168 | SLC30A7 |
| 12178 | SLC35A4 |
| 12207 | SLC38A11 |
| 12218 | SLC39A13 |
| 12238 | SLC45A1 |
| 12243 | SLC47A1 |
| 12250 | SLC4A3 |
| 12270 | SLC6A15 |
| 12275 | SLC6A6 |
| 12276 | SLC6A7 |
| 12281 | SLC7A14 |
| 12283 | SLC7A4 |
| 12290 | SLC8A1 |
| 12319 | SLIT3 |
| 12322 | SLITRK3 |
| 12356 | SMARCD2 |
| 12366 | SMCO2 |
| 12379 | SMIM10 |
| 12381 | SMIM10L2A |
| 12382 | SMIM10L2B |
| 12386 | SMIM14 |
| 12394 | SMIM29 |
| 12412 | SMPDL3A |
| 12413 | SMPX |
| 12437 | SNAPC5 |
| 12439 | SNCA |
| 12440 | SNCAIP |
| 12442 | SNCG |
| 12491 | SNTG1 |
| 12492 | SNTG2 |
| 12534 | SOD1 |
| 12539 | SOHLH1 |
| 12541 | SORBS1 |
| 12544 | SORCS1 |
| 12553 | SOWAHA |
| 12589 | SPACA3 |
| 12593 | SPAG4 |
| 12599 | SPARC |
| 12655 | SPINT2 |
| 12661 | SPNS3 |
| 12705 | SPTSSB |
| 12708 | SQLE |
| 12718 | SRD5A3 |
| 12733 | SRP14 |
| 12740 | SRPK1 |
| 12753 | SRSF1 |
| 12791 | SSTR1 |
| 12792 | SSTR2 |
| 12826 | ST8SIA5 |
| 12828 | STAC2 |
| 12844 | STARD3NL |
| 12846 | STARD5 |
| 12864 | STEAP2 |
| 12879 | STK26 |
| 12886 | STK38L |
| 12898 | STOML1 |
| 12909 | STRBP |
| 12912 | STRN |
| 12921 | STX12 |
| 12924 | STX17-AS1 |
| 12927 | STX19 |
| 12955 | SUGP2 |
| 12960 | SULF2 |
| 12989 | SUSD3 |
| 13014 | SYCP2 |
| 13017 | SYDE2 |
| 13026 | SYNDIG1 |
| 13058 | SYT17 |
| 13066 | SYTL2 |
| 13099 | TAF1C |
| 13131 | TAPBP |
| 13137 | TARDBP |
| 13143 | TAS2R10 |
| 13151 | TAS2R4 |
| 13165 | TBC1D1 |
| 13173 | TBC1D16 |
| 13181 | TBC1D24 |
| 13195 | TBC1D8B |
| 13198 | TBCA |
| 13215 | TBR1 |
| 13227 | TC2N |
| 13234 | TCEA3 |
| 13257 | TCF7L2 |
| 13261 | TCIM |
| 13294 | TECTA |
| 13315 | TESC |
| 13318 | TESPA1 |
| 13327 | TEX29 |
| 13328 | TEX30 |
| 13330 | TEX47 |
| 13343 | TFEC |
| 13380 | THAP8 |
| 13386 | THBS3 |
| 13405 | THSD4 |
| 13416 | TIAM2 |
| 13417 | TICAM1 |
| 13442 | TIMM8B |
| 13446 | TIMP2 |
| 13461 | TKFC |
| 13474 | TLL1 |
| 13498 | TM7SF3 |
| 13561 | TMEM132D |
| 13581 | TMEM155 |
| 13583 | TMEM159 |
| 13587 | TMEM161B-AS1 |
| 13595 | TMEM17 |
| 13602 | TMEM176A |
| 13603 | TMEM176B |
| 13627 | TMEM200A |
| 13668 | TMEM255A |
| 13708 | TMEM61 |
| 13715 | TMEM67 |
| 13726 | TMEM86B |
| 13755 | TMSB15B |
| 13770 | TNFAIP1 |
| 13771 | TNFAIP2 |
| 13786 | TNFRSF25 |
| 13804 | TNNC2 |
| 13808 | TNNT2 |
| 13859 | TOX |
| 13860 | TOX2 |
| 13866 | TP53I11 |
| 13896 | TPP2 |
| 13907 | TPST2 |
| 13969 | TRIB3 |
| 13985 | TRIM27 |
| 13991 | TRIM35 |
| 14036 | TRMT112 |
| 14047 | TRMT9B |
| 14056 | TRPC3 |
| 14060 | TRPM2 |
| 14069 | TRPV2 |
| 14075 | TSACC |
| 14097 | TSNAXIP1 |
| 14116 | TSPAN9 |
| 14137 | TTC1 |
| 14138 | TTC12 |
| 14179 | TTLL11 |
| 14188 | TTPAL |
| 14196 | TUBA1A |
| 14205 | TUBB |
| 14206 | TUBB2A |
| 14207 | TUBB2B |
| 14208 | TUBB3 |
| 14210 | TUBB4B |
| 14211 | TUBB6 |
| 14213 | TUBB8P12 |
| 14214 | TUBD1 |
| 14236 | TWIST1 |
| 14244 | TXN |
| 14264 | TYROBP |
| 14280 | UBA52 |
| 14313 | UBE2L5 |
| 14317 | UBE2N |
| 14346 | UBN2 |
| 14362 | UBXN10 |
| 14369 | UBXN8 |
| 14370 | UCHL1 |
| 14379 | UCP3 |
| 14395 | UHMK1 |
| 14464 | USF2 |
| 14479 | USP2 |
| 14547 | VAMP8 |
| 14549 | VANGL2 |
| 14559 | VAT1L |
| 14560 | VAV1 |
| 14585 | VILL |
| 14589 | VIPR1 |
| 14590 | VIPR2 |
| 14643 | VSIG10L |
| 14650 | VSTM2B |
| 14658 | VWA3A |
| 14659 | VWA5A |
| 14663 | VWC2 |
| 14670 | WAPL |
| 14694 | WDFY4 |
| 14697 | WDR1 |
| 14711 | WDR33 |
| 14767 | WFS1 |
| 14784 | WNT10A |
| 14785 | WNT10B |
| 14792 | WNT6 |
| 14793 | WNT7A |
| 14805 | WTIP |
| 14820 | XK |
| 14822 | XKR6 |
| 14846 | XYLT2 |
| 14863 | YIPF1 |
| 14873 | YOD1 |
| 14889 | YWHAH |
| 14897 | ZADH2 |
| 14900 | ZBBX |
| 14904 | ZBED5 |
| 14914 | ZBTB16 |
| 14919 | ZBTB21 |
| 14928 | ZBTB38 |
| 14941 | ZBTB7A |
| 14953 | ZC3H12C |
| 14964 | ZC3HAV1 |
| 14970 | ZCCHC12 |
| 14972 | ZCCHC17 |
| 14973 | ZCCHC18 |
| 14980 | ZCCHC8 |
| 14987 | ZDHHC12 |
| 15007 | ZDHHC8P1 |
| 15022 | ZFHX2 |
| 15037 | ZFP64 |
| 15061 | ZHX1 |
| 15065 | ZIC3 |
| 15073 | ZKSCAN4 |
| 15094 | ZMYND15 |
| 15096 | ZMYND8 |
| 15108 | ZNF133 |
| 15120 | ZNF148 |
| 15146 | ZNF211 |
| 15165 | ZNF232 |
| 15182 | ZNF260 |
| 15187 | ZNF268 |
| 15201 | ZNF284 |
| 15242 | ZNF362 |
| 15243 | ZNF365 |
| 15251 | ZNF385B |
| 15252 | ZNF385D |
| 15258 | ZNF398 |
| 15289 | ZNF444 |
| 15312 | ZNF487 |
| 15313 | ZNF488 |
| 15334 | ZNF519 |
| 15335 | ZNF521 |
| 15344 | ZNF534 |
| 15358 | ZNF555 |
| 15430 | ZNF646 |
| 15459 | ZNF684 |
| 15518 | ZNF77 |
| 15543 | ZNF8 |

**Table SI 5: Functional enrichment results of the genes associated with sleep status × rTMS intervention ANOVA**

| **Category** | **Pathway** | **GeneRatio** | **FDR** |
| --- | --- | --- | --- |
| GO: Molecular Function | voltage-gated cation channel activity | 2.80E-02 | 1.66E-05 |
| GO: Molecular Function | gated channel activity | 4.46E-02 | 3.74E-05 |
| GO: Molecular Function | voltage-gated potassium channel activity | 2.07E-02 | 3.74E-05 |
| GO: Molecular Function | voltage-gated ion channel activity | 3.11E-02 | 3.74E-05 |
| GO: Molecular Function | voltage-gated channel activity | 3.11E-02 | 3.74E-05 |
| GO: Molecular Function | metal ion transmembrane transporter activity | 4.97E-02 | 1.39E-04 |
| GO: Molecular Function | potassium channel activity | 2.18E-02 | 1.70E-04 |
| GO: Molecular Function | ion channel activity | 4.97E-02 | 1.99E-04 |
| GO: Molecular Function | potassium ion transmembrane transporter activity | 2.49E-02 | 2.44E-04 |
| GO: Molecular Function | neurotransmitter receptor activity | 1.97E-02 | 4.08E-04 |
| GO: Molecular Function | cation channel activity | 4.04E-02 | 4.50E-04 |
| GO: Molecular Function | channel activity | 4.97E-02 | 1.89E-03 |
| GO: Molecular Function | passive transmembrane transporter activity | 4.97E-02 | 1.89E-03 |
| GO: Molecular Function | postsynaptic neurotransmitter receptor activity | 1.45E-02 | 1.89E-03 |
| GO: Molecular Function | delayed rectifier potassium channel activity | 9.33E-03 | 2.08E-03 |
| GO: Molecular Function | neurotransmitter receptor activity involved in regulation of postsynaptic membrane potential | 1.24E-02 | 3.55E-03 |
| GO: Molecular Function | ligand-gated ion channel activity | 2.07E-02 | 3.74E-03 |
| GO: Molecular Function | ligand-gated channel activity | 2.07E-02 | 3.74E-03 |
| GO: Molecular Function | PDZ domain binding | 1.45E-02 | 3.89E-03 |
| GO: Molecular Function | extracellular ligand-gated ion channel activity | 1.35E-02 | 4.86E-03 |
| GO: Molecular Function | transmitter-gated ion channel activity | 1.24E-02 | 4.92E-03 |
| GO: Molecular Function | transmitter-gated channel activity | 1.24E-02 | 4.92E-03 |
| GO: Molecular Function | GABA-A receptor activity | 6.22E-03 | 1.07E-02 |
| GO: Molecular Function | ligand-gated anion channel activity | 6.22E-03 | 1.07E-02 |
| GO: Molecular Function | nuclear receptor activity | 1.04E-02 | 1.07E-02 |
| GO: Molecular Function | ligand-activated transcription factor activity | 1.04E-02 | 1.07E-02 |
| GO: Molecular Function | GABA-gated chloride ion channel activity | 5.18E-03 | 1.16E-02 |
| GO: Molecular Function | structural constituent of cytoskeleton | 1.55E-02 | 1.43E-02 |
| GO: Molecular Function | channel regulator activity | 1.87E-02 | 1.77E-02 |
| GO: Molecular Function | serine hydrolase activity | 2.28E-02 | 1.84E-02 |
| GO: Molecular Function | GABA receptor activity | 6.22E-03 | 2.09E-02 |
| GO: Molecular Function | inhibitory extracellular ligand-gated ion channel activity | 5.18E-03 | 2.09E-02 |
| GO: Molecular Function | transmitter-gated ion channel activity involved in regulation of postsynaptic membrane potential | 1.04E-02 | 2.09E-02 |
| GO: Molecular Function | DNA-binding transcription repressor activity, RNA polymerase II-specific | 3.21E-02 | 2.25E-02 |
| GO: Molecular Function | ion channel regulator activity | 1.76E-02 | 2.60E-02 |
| GO: Molecular Function | DNA-binding transcription repressor activity | 3.21E-02 | 2.60E-02 |
| GO: Molecular Function | serine-type peptidase activity | 2.18E-02 | 2.63E-02 |
| GO: Molecular Function | nuclear steroid receptor activity | 6.22E-03 | 2.63E-02 |
| GO: Molecular Function | dipeptidyl-peptidase activity | 4.15E-03 | 2.63E-02 |
| GO: Molecular Function | protein tyrosine/threonine phosphatase activity | 4.15E-03 | 2.63E-02 |
| GO: Molecular Function | MHC class II receptor activity | 4.15E-03 | 2.63E-02 |
| GO: Molecular Function | calmodulin binding | 2.18E-02 | 4.02E-02 |
| GO: Molecular Function | MHC protein complex binding | 7.25E-03 | 4.62E-02 |
| GO: Molecular Function | MHC class II protein complex binding | 6.22E-03 | 4.62E-02 |
| GO: Molecular Function | excitatory extracellular ligand-gated ion channel activity | 9.33E-03 | 4.62E-02 |
| GO: Biological Process | regulation of membrane potential | 5.07E-02 | 9.26E-04 |
| GO: Biological Process | signal release | 5.29E-02 | 1.05E-03 |
| GO: Biological Process | rhythmic process | 3.70E-02 | 4.54E-03 |
| GO: Biological Process | regulation of peptide secretion | 2.64E-02 | 4.54E-03 |
| GO: Biological Process | synapse assembly | 2.64E-02 | 4.54E-03 |
| GO: Biological Process | regulation of peptide transport | 2.64E-02 | 4.54E-03 |
| GO: Biological Process | regulation of peptide hormone secretion | 2.54E-02 | 7.50E-03 |
| GO: Biological Process | regulation of circadian rhythm | 1.90E-02 | 1.55E-02 |
| GO: Biological Process | peptide transport | 3.07E-02 | 1.55E-02 |
| GO: Biological Process | regulation of cell junction assembly | 2.64E-02 | 1.55E-02 |
| GO: Biological Process | amide transport | 3.38E-02 | 1.55E-02 |
| GO: Biological Process | regulation of postsynaptic membrane potential | 2.11E-02 | 1.55E-02 |
| GO: Biological Process | circadian rhythm | 2.64E-02 | 2.04E-02 |
| GO: Biological Process | cell junction assembly | 4.33E-02 | 2.04E-02 |
| GO: Biological Process | neurotransmitter transport | 2.64E-02 | 2.04E-02 |
| GO: Biological Process | regulation of neurotransmitter transport | 1.69E-02 | 2.04E-02 |
| GO: Biological Process | regulation of trans-synaptic signalling | 4.44E-02 | 2.26E-02 |
| GO: Biological Process | protein homooligomerization | 2.43E-02 | 2.26E-02 |
| GO: Biological Process | regulation of metal ion transport | 4.23E-02 | 2.30E-02 |
| GO: Biological Process | peptide secretion | 2.75E-02 | 2.30E-02 |
| GO: Biological Process | regulation of cell morphogenesis | 3.38E-02 | 2.39E-02 |
| GO: Biological Process | potassium ion transport | 2.85E-02 | 2.55E-02 |
| GO: Biological Process | protein complex oligomerization | 2.85E-02 | 2.55E-02 |
| GO: Biological Process | regulation of hormone secretion | 2.75E-02 | 2.55E-02 |
| GO: Biological Process | response to ATP | 8.46E-03 | 2.73E-02 |
| GO: Biological Process | potassium ion transmembrane transport | 2.64E-02 | 2.73E-02 |
| GO: Biological Process | modulation of chemical synaptic transmission | 4.33E-02 | 2.77E-02 |
| GO: Biological Process | positive regulation of secretion by cell | 3.07E-02 | 2.79E-02 |
| GO: Biological Process | peptide hormone secretion | 2.64E-02 | 2.79E-02 |
| GO: Biological Process | regulation of insulin secretion | 2.01E-02 | 2.79E-02 |
| GO: Biological Process | circadian regulation of gene expression | 1.27E-02 | 3.13E-02 |
| GO: Biological Process | regulation of synapse assembly | 1.59E-02 | 3.21E-02 |
| GO: Biological Process | response to acetylcholine | 8.46E-03 | 3.31E-02 |
| GO: Biological Process | actomyosin structure organization | 2.43E-02 | 3.45E-02 |
| GO: Biological Process | cardiac myofibril assembly | 6.34E-03 | 3.45E-02 |
| GO: Biological Process | positive regulation of synapse assembly | 1.16E-02 | 3.60E-02 |
| GO: Biological Process | regulation of neuron projection development | 4.23E-02 | 3.60E-02 |
| GO: Biological Process | synapse organization | 4.12E-02 | 3.60E-02 |
| GO: Biological Process | excitatory synapse assembly | 7.40E-03 | 3.60E-02 |
| GO: Biological Process | regulation of excitatory synapse assembly | 5.29E-03 | 3.60E-02 |
| GO: Biological Process | MHC protein complex assembly | 6.34E-03 | 3.77E-02 |
| GO: Biological Process | peptide antigen assembly with MHC protein complex | 6.34E-03 | 3.77E-02 |
| GO: Biological Process | postsynaptic specialization assembly | 6.34E-03 | 3.77E-02 |
| GO: Biological Process | regulation of synapse organization | 2.43E-02 | 3.77E-02 |
| GO: Biological Process | positive regulation of synaptic transmission | 2.11E-02 | 3.77E-02 |
| GO: Biological Process | regulation of ossification | 1.69E-02 | 4.22E-02 |
| GO: Biological Process | positive regulation of biomineralization | 1.06E-02 | 4.40E-02 |
| GO: Biological Process | regulation of nervous system development | 4.23E-02 | 4.45E-02 |
| GO: Biological Process | negative regulation of glucose transmembrane transport | 6.34E-03 | 4.52E-02 |
| GO: Biological Process | postsynapse assembly | 7.40E-03 | 4.58E-02 |
| GO: Biological Process | excitatory postsynaptic potential | 1.69E-02 | 4.58E-02 |
| GO: Biological Process | regulation of synapse structure or activity | 2.43E-02 | 4.84E-02 |
| GO: Biological Process | glucose transmembrane transport | 1.59E-02 | 4.84E-02 |
| GO: Biological Process | positive regulation of insulin secretion | 1.16E-02 | 4.86E-02 |
| GO: Cellular Component | synaptic membrane | 4.85E-02 | 1.46E-06 |
| GO: Cellular Component | postsynaptic membrane | 3.84E-02 | 1.80E-06 |
| GO: Cellular Component | ion channel complex | 4.04E-02 | 1.80E-06 |
| GO: Cellular Component | voltage-gated potassium channel complex | 1.82E-02 | 6.70E-06 |
| GO: Cellular Component | potassium channel complex | 1.92E-02 | 7.01E-06 |
| GO: Cellular Component | cation channel complex | 3.13E-02 | 1.96E-05 |
| GO: Cellular Component | integral component of synaptic membrane | 2.42E-02 | 5.26E-05 |
| GO: Cellular Component | intrinsic component of synaptic membrane | 2.53E-02 | 5.26E-05 |
| GO: Cellular Component | transporter complex | 4.44E-02 | 6.66E-05 |
| GO: Cellular Component | transmembrane transporter complex | 4.14E-02 | 1.55E-04 |
| GO: Cellular Component | integral component of postsynaptic membrane | 1.92E-02 | 2.96E-04 |
| GO: Cellular Component | intrinsic component of postsynaptic membrane | 1.92E-02 | 5.03E-04 |
| GO: Cellular Component | glutamatergic synapse | 3.43E-02 | 1.55E-03 |
| GO: Cellular Component | GABA-ergic synapse | 1.31E-02 | 2.00E-03 |
| GO: Cellular Component | MHC class II protein complex | 6.06E-03 | 4.36E-03 |
| GO: Cellular Component | intercellular bridge | 1.41E-02 | 4.36E-03 |
| GO: Cellular Component | neuronal cell body | 4.44E-02 | 5.56E-03 |
| GO: Cellular Component | postsynaptic specialization membrane | 1.62E-02 | 5.82E-03 |
| GO: Cellular Component | Schaffer collateral - CA1 synapse | 1.21E-02 | 6.31E-03 |
| GO: Cellular Component | GABA-A receptor complex | 6.06E-03 | 6.61E-03 |
| GO: Cellular Component | integral component of postsynaptic specialization membrane | 1.21E-02 | 9.60E-03 |
| GO: Cellular Component | presynaptic membrane | 1.82E-02 | 1.06E-02 |
| GO: Cellular Component | GABA receptor complex | 6.06E-03 | 1.06E-02 |
| GO: Cellular Component | dendritic spine | 2.02E-02 | 1.08E-02 |
| GO: Cellular Component | postsynaptic specialization | 3.23E-02 | 1.08E-02 |
| GO: Cellular Component | neuron spine | 2.02E-02 | 1.08E-02 |
| GO: Cellular Component | intrinsic component of postsynaptic specialization membrane | 1.21E-02 | 1.08E-02 |
| GO: Cellular Component | MHC protein complex | 6.06E-03 | 2.38E-02 |
| GO: Cellular Component | postsynaptic density | 2.93E-02 | 2.76E-02 |
| GO: Cellular Component | postsynaptic density membrane | 1.21E-02 | 2.76E-02 |
| GO: Cellular Component | calyx of Held | 5.05E-03 | 2.76E-02 |
| GO: Cellular Component | axon terminus | 1.41E-02 | 3.08E-02 |
| GO: Cellular Component | asymmetric synapse | 2.93E-02 | 3.32E-02 |
| GO: Cellular Component | intrinsic component of presynaptic membrane | 1.11E-02 | 3.32E-02 |
| GO: Cellular Component | neuron projection terminus | 1.52E-02 | 3.63E-02 |
| GO: Cellular Component | neuron projection membrane | 9.09E-03 | 4.30E-02 |
| GO: Cellular Component | integral component of presynaptic membrane | 1.01E-02 | 4.86E-02 |
| GO: Cellular Component | distal axon | 2.53E-02 | 4.86E-02 |
| GO: Cellular Component | anchored component of plasma membrane | 9.09E-03 | 4.94E-02 |
| GO: Cellular Component | integral component of postsynaptic density membrane | 8.08E-03 | 4.94E-02 |
| KEGG: Disease | Neuroactive ligand‒receptor interaction | 9.93E-02 | 2.13E-05 |
| KEGG: Disease | Gap junction | 3.97E-02 | 8.04E-05 |
| KEGG: Disease | cAMP signalling pathway | 6.18E-02 | 1.76E-03 |
| KEGG: Disease | Morphine addiction | 3.31E-02 | 5.32E-03 |
| KEGG: Disease | Motor proteins | 5.08E-02 | 9.57E-03 |
| KEGG: Disease | Inflammatory mediator regulation of TRP channels | 3.09E-02 | 1.81E-02 |
| KEGG: Disease | Pathways of neurodegeneration - multiple diseases | 9.27E-02 | 1.81E-02 |
| KEGG: Disease | Melanogenesis | 3.09E-02 | 1.81E-02 |
| KEGG: Disease | Calcium signalling pathway | 5.74E-02 | 1.81E-02 |
| KEGG: Disease | Nicotine addiction | 1.77E-02 | 2.06E-02 |
| KEGG: Disease | Cognitive impairment | 2.87E-02 | 2.96E-02 |
| KEGG: Disease | Circadian rhythm | 1.55E-02 | 3.14E-02 |
| KEGG: Disease | Phagosome | 3.75E-02 | 4.29E-02 |
| KEGG: Disease | cGMP-PKG signalling pathway | 3.97E-02 | 4.63E-02 |

**Table SI 6. Hub genes**

| **Gene symbol** | **Betweenness** |
| --- | --- |
| MAPK3 | 45439.42 |
| CDC42 | 33399.957 |
| PRKCB | 23455.016 |
| UBA52 | 22274.629 |
| IL1B | 21486.129 |
| SOD1 | 19473.135 |
| GRIN2A | 19265.943 |
| PPARGC1A | 16164.019 |
| LRRK2 | 14344.084 |
| HCLS1 | 12881.585 |
| TUBA1A | 12719.565 |
| CEBPB | 12558.796 |
| PTPRC | 11342.031 |
| VAV1 | 11105.927 |
| HLA-DRB1 | 10681.234 |
| PTK2B | 10469.161 |
| CD74 | 10401.925 |
| GFAP | 9923.974 |
| PTGS2 | 9680.256 |
| CD8A | 9488.264 |
| RPH3A | 9458 |
| NR3C1 | 9091.607 |
| ENO1 | 8671.095 |
| GNG7 | 8314.353 |
| TUBB | 8192.584 |
| KCNA2 | 8077.6934 |
| RAB27B | 7358 |
| RAC2 | 7228.737 |
| PPP1CB | 7137.6074 |
| PSMB2 | 6872.263 |
| EGR1 | 6539.1426 |
| PLCB1 | 6347.9463 |
| AEBP2 | 6268.381 |
| PTGIS | 5872 |
| CDKN2A | 5592.631 |
| KNG1 | 5578.611 |
| TARDBP | 5460.1846 |
| UCHL1 | 5358.026 |
